# Developmental patterns of affective attention across the first 2 years of life

**DOI:** 10.1111/cdev.13831

**Published:** 2022-07-29

**Authors:** Lori B. Reider, Laura Bierstedt, Jessica L. Burris, Alicia Vallorani, Kelley E. Gunther, Kristin A. Buss, Koraly Pérez‐Edgar, Andy P. Field, Vanessa LoBue

**Affiliations:** ^1^ Department of Psychology Rutgers University Newark New Jersey USA; ^2^ Department of Psychology Pennsylvania State University State College Pennsylvania USA; ^3^ Department of Psychology Yale University New Haven Connecticut USA; ^4^ School of Psychology University of Sussex Brighton UK

## Abstract

This study examined patterns of attention toward affective stimuli in a longitudinal sample of typically developing infants (*N* = 357, 147 females, 50% White, 22% Latinx, 16% African American/Black, 3% Asian, 8% mixed race, 1% not reported) using two eye‐tracking tasks that measure vigilance to (rapid detection), engagement with (total looking toward), and disengagement from (latency to looking away) emotional facial configurations. Infants completed each task at 4, 8, 12, 18, and 24 months of age from 2016 to 2020. Multilevel growth models demonstrate that, over the first 2 years of life, infants became faster at detecting and spent more time engaging with angry over neutral faces. These results have implications for our understanding of the development of affect‐biased attention.

**Abbreviations**
AOI: area of interest
CI: confidence interval
ML: maximum likelihood
REML: restricted maximum likelihood
SD: standard deviation

The visual attention system is fundamental for information processing, early learning, and socioemotional development (Morales et al., 2016; Posner & Rothbart, 2007). Visual attention plays an important role in infants' ability to perceive and interpret the emotional facial expressions of others. Indeed, research suggests that infants display a visual preference toward face‐like stimuli from birth (Johnson et al., 1991; Pascalis & Kelly, 2009; Valenza et al., 1996), and by the second half of the first year of life, infants can discriminate between emotional facial configurations based on positive and negative valence (Barrera & Maurer, 1981; Farroni et al., 2007).

Affect‐biased attention—or the prioritization of affective or motivationally salient information in visual attention—is also evident early in life (Morales et al., 2016; Todd et al., 2012). One specific form of affect‐biased attention that has been widely explored in the literature is attention bias to threat, commonly defined as rapid detection of, or slower disengagement from, threat‐relevant stimuli over neutral or positive stimuli (Amso & Scerif, 2015; Cisler & Koster, 2010). Threatening stimuli in this literature typically include emotional expressions such as angry facial configurations, which are a direct sign of threat, fearful facial configurations, which are an indirect sign of threat, as well as threatening animals such as snakes and spiders (Adams & Kleck, 2003; Morales et al., 2017). While attention biases for threat, particularly for angry facial configurations, are viewed in the clinical literature as a risk factor for the development of anxiety disorders in both adults and children (Abend et al., 2018; Armstrong & Olatunji, 2012; Bar‐Haim et al., 2007), other researchers have suggested that such biases to threat are normative and evolutionarily adaptive, functioning to help humans quickly respond to potentially threatening encounters (Öhman & Mineka, 2001). In support of this perspective, several studies have reported that adults detect threatening stimuli faster than benign control stimuli (see Burris et al., 2019, for a review). In addition, attention biases to threat typically emerge early in development (Peltola, Leppänen, Mäki, et al., 2009; Peltola, Leppänen, Vogel‐Farley, et al., 2009). Specifically, by 6–8 months of age, infants look longer at fearful and angry facial configurations (e.g., Leppänen et al., 2018; Morales et al., 2016; Peltola et al., 2018), show greater difficulty disengaging from fearful facial configurations (e.g., Peltola et al., 2008, 2013; Peltola, Leppänen, Vogel‐Farley, et al., 2009), and are faster to detect fearful and angry facial configurations when compared to positive or neutral stimuli (e.g., LoBue & DeLoache, 2010; Nakagawa & Sukigara, 2019).

However, not all findings from this literature are consistent. For example, while some studies report faster detection (e.g., LoBue & DeLoache, 2010), or slower disengagement from threat in infants (e.g., Nakagawa & Sukigara, 2012; Peltola et al., 2008; Peltola, Leppänen, Vogel‐Farley, et al., 2009), other studies suggest that infants have a bias for *positive* emotional stimuli (Grossmann et al., 2007; LaBarbera et al., 1976; Wilcox & Clayton, 1968). Furthermore, studies have also documented that infants demonstrate a bias for *any* emotional information, including both positive and negative emotional facial configurations (Burris et al., 2017).

One potential explanation for these inconsistent findings is that attention biases to threat and other emotionally valenced stimuli develop and change over time. Moreover, while some attention biases are normative and early developing, others might diverge from these normative trajectories to become markers of risk for psychopathology at some point in development. Field and Lester (2010) have proposed several potential models for how attention biases to threat might emerge over the first few years of life. The integral‐bias model suggests that attention biases to threat are present early in life and remain stable across development. The moderation model posits that attention biases to threat are normative early in life, but certain circumstances across development moderate the link between attention biases and anxiety later in life. Finally, the acquisition model posits that attention biases to threat are not present early in life, but instead, may emerge over time after an individual experiences a specific event or are exposed to environments that cause the bias to develop. Unfortunately, to date, developmental research on this topic is still quite limited, and studies that do exist with infants mostly use cross‐sectional designs, especially early in life. This work can provide only a snapshot of attention biases to threat at a single point in time, and we still know very little about the emergence and developmental trajectories of attention biases over time.

In addition to the role of development, another potential explanation for the discrepant findings in the literature is that studies on attention biases to threat rely on different tasks that are designed to tap into different components of attention. The visual attention system comprises several interacting neural networks including the alerting, orienting, and executive attention networks (Corbetta & Shulman, 2002; Petersen & Posner, 2012; Rothbart et al., 2011). The role of the *alerting* network is to detect information in the visual field, and it is responsible for selective attention and detection of novel stimuli (Sturm & Willmes, 2001). The *orienting* network is responsible for the selection and prioritization of information in the environment, including disengaging, shifting, and re‐engaging with visual stimuli (Posner et al., 1984). Finally, the *executive attention* network is responsible for more voluntary control and regulation of attention processing, including working memory, set shifting, and inhibition (Rothbart et al., 2011). The alerting and orienting networks are primarily stimulus‐driven and reflect a bottom‐up processing that develops early in infancy (Corbetta & Shulman, 2002; Rothbart et al., 2011), and the executive attention network relies on top‐down processes and typically develops by the second year of life (Rothbart et al., 2011).

While all three systems are functional early in life and are assessed in the affect‐biased attention literature, little work has attempted to differentiate the developmental trajectories of each component of attention bias to threat, especially in the alerting and orienting networks that should be fully developed in infancy. Furthermore, while the rapid detection of threatening stimuli (e.g., alerting), as well as engagement to and disengagement from threatening stimuli (e.g., orienting) may reflect automatic, exogenously driven processes, there is little work empirically examining whether they follow similar or unique trajectories. In addition, we do not know if patterns in alerting and orienting uniquely impact socioemotional development. Such distinctions in attention processing beg the question of whether different components of affect‐biased attention reflect similar or distinct patterns of emotion processing, and whether a bias for threat emerges differently across different components of attention.

Here, we asked two important questions. First, are attention biases for threat—specifically the prioritization of angry facial configurations over neutral ones—evident early in life in a sample of typically developing infants? And second, is the emergence of these potentially normative biases different across different components of attention? To answer these questions, we tested a large, longitudinal sample of infants using two eye‐tracking tasks at 4, 8, 12, 18, and 24 months of age. Each task was designed to tap into different components of affective attention.

In the vigilance task (Fu et al., 2020), which taps into the alerting network, we aimed to measure infants' rapid detection of threat. Infants were shown a single stimulus—either a happy, angry, or neutral facial configuration—randomly presented in one of the four corners of a screen on each trial, and we measured how quickly infants first fixated, or detected, each target. Rapid detection was indexed by time to first fixation to angry, happy, or neutral facial configurations. Here, an attention bias to threat would be demonstrated by faster detection of angry facial configurations than neutral ones.

In the overlap task (i.e., Morales et al., 2017; Peltola et al., 2008), which taps into the orienting network, we aimed to measure both engagement and disengagement from threat. We presented infants with a happy, angry, or neutral facial configuration in the center of a screen, and shortly after their appearance, a checkerboard probe appeared simultaneously to the left or the right of the facial stimulus. To index engagement, we measured total looking to the facial stimuli while a probe was also on the screen. In this task, infants would demonstrate an attention bias to threat by more engagement with or longer looking at angry versus neutral facial stimuli in the presence of the probe. To index disengagement, we measured latency to fixate the probe which appeared shortly after the presence of an emotional facial configuration. Here, an attention bias to threat would be demonstrated by less disengagement from threatening facial stimuli, defined as longer latencies to fixate the probe on angry versus neutral trials. Previous work supports the idea that engagement with threatening stimuli and disengagement from threatening stimuli in this task and represents unique components of affect‐biased attention (Vallorani et al., 2021).

In summary, our first aim was to explore and describe the change in attention toward emotional facial stimuli across the first 2 years of life among different components of attention. To do this, we ran a series of exploratory analyses using multilevel growth models for rapid detection, engagement, and disengagement to compare angry (threat) and happy facial configurations to neutral ones. Our second aim was to identify when a bias to threat (in this case, angry facial configurations) first emerges across different components of attention. To address this aim, we ran additional exploratory analyses using paired samples *t*‐tests to determine when an attention bias for threat was present. To explore whether these biases are specific to threat, or develop for all emotions more broadly, we also aimed to explore whether a bias for positive stimuli was present by comparing attention to happy facial configurations with neutral ones at each assessment across tasks.

## METHOD

### Participants

The participants and methodology described here were part of a larger study (*N* = 357) examining the development of attention and temperament across the first 2 years of life (Pérez‐Edgar et al., 2021). Participants were recruited through local baby registries (40%), university‐sponsored participant databases (13%), community‐level recruitment strategies (38%), and word‐of‐mouth (10%). The Institutional Review Boards at Pennsylvania State University and Rutgers University approved all procedures and parents provided written consent and were compensated for their participation.

Infants and their caregivers were enrolled when the infants were 4 months of age (*N* = 298; 147 females), with an additional 46 participants enrolled at 8 months (*N* = 46; 27 females), and 13 participants at 12 months (*N* = 13; 7 females), for a total enrollment of 357 infants in the full sample (176 males, 181 females). Participants were recruited from areas surrounding three sites within the United States: State College, PA (*N* = 167), Harrisburg, PA (*N* = 81), and Newark, NJ (*N* = 109). All the data used in the following analyses were collected in‐person between November 2016 and March 2020, prior to the onset of the COVID‐19 pandemic, and completion of data collection at the 8‐, 12‐, 18‐, and 24‐month assessments were impacted by the COVID‐19 pandemic. Additional demographic information is provided in Table 1.

**TABLE 1:** Demographic information

Infant ages										
	4‐Month assessment		8‐Month assessment		12‐Month assessment		18‐Month assessment		24‐Month assessment	
	*M*	*SD*	*M*	*SD*	*M*	*SD*	*M*	*SD*	*M*	*SD*
Age at eye‐tracking	4.83	0.82	8.38	1.08	12.40	1.10	18.36	0.78	24.64	1.15

Infant race/ethnicity					
African American/Black	Asian	Latinx	White	Mixed race	Not reported
58 (16%)	9 (3%)	78 (22%)	180 (50%)	27 (8%)	5 (1%)

Household income at enrollment							
≤$15,000	$16,000–$20,000	$21,000–$30,000	$31,000–$40,000	$41,000–$50,000	$51,000–$60,000	Above $60,000	Not reported
49 (14%)	20 (6%)	22 (6%)	16 (5%)	22 (6%)	29 (8%)	140 (39%)	59 (17%)

Mother's education at enrollment							
Grade school	Some high school	High school graduate	Some college or trade/technical degree	College graduate	Graduate training	Graduate degree	Not reported
11 (3%)	17 (5%)	36 (10%)	57 (16%)	73 (20%)	58 (16%)	66 (19%)	39 (11%)

Father's education at enrollment							
Grade school	Some high school	High school graduate	Some college or trade/technical degree	College graduate	Graduate training	Graduate gegree	Not reported
11 (3%)	15 (4%)	50 (14%)	60 (17%)	70 (20%)	42 (12%)	56 (16%)	53 (15%)

### Eye‐tracking tasks

Eye‐tracking data were collected across sites using SMI eye tracking systems, either the SMI RED or REDm system, both offering comparable specifications and capabilities (SensoMotoric Instruments). Participants were seated ~60 cm from a 22′′ Dell monitor for stimulus presentation, in a highchair. If needed, infants could also sit on their parent's lap or on the lap of an experimenter. Gaze was calibrated using a 5‐point calibration followed by a 4‐point validation, using an animated flower on a black screen and infant‐friendly music. Gaze data were sampled at 60 Hz and collected by Experiment Center (SensoMotoric Instruments). Infants were calibrated below 4° of visual angle from all calibration points. Infants completed three eye tracking tasks: the vigilance, overlap, and infant dot‐probe tasks (note the Dot Probe was not examined in this analysis). Order of task presentation was randomized prior to the visits.

### Vigilance task

**FIGURE 1:**
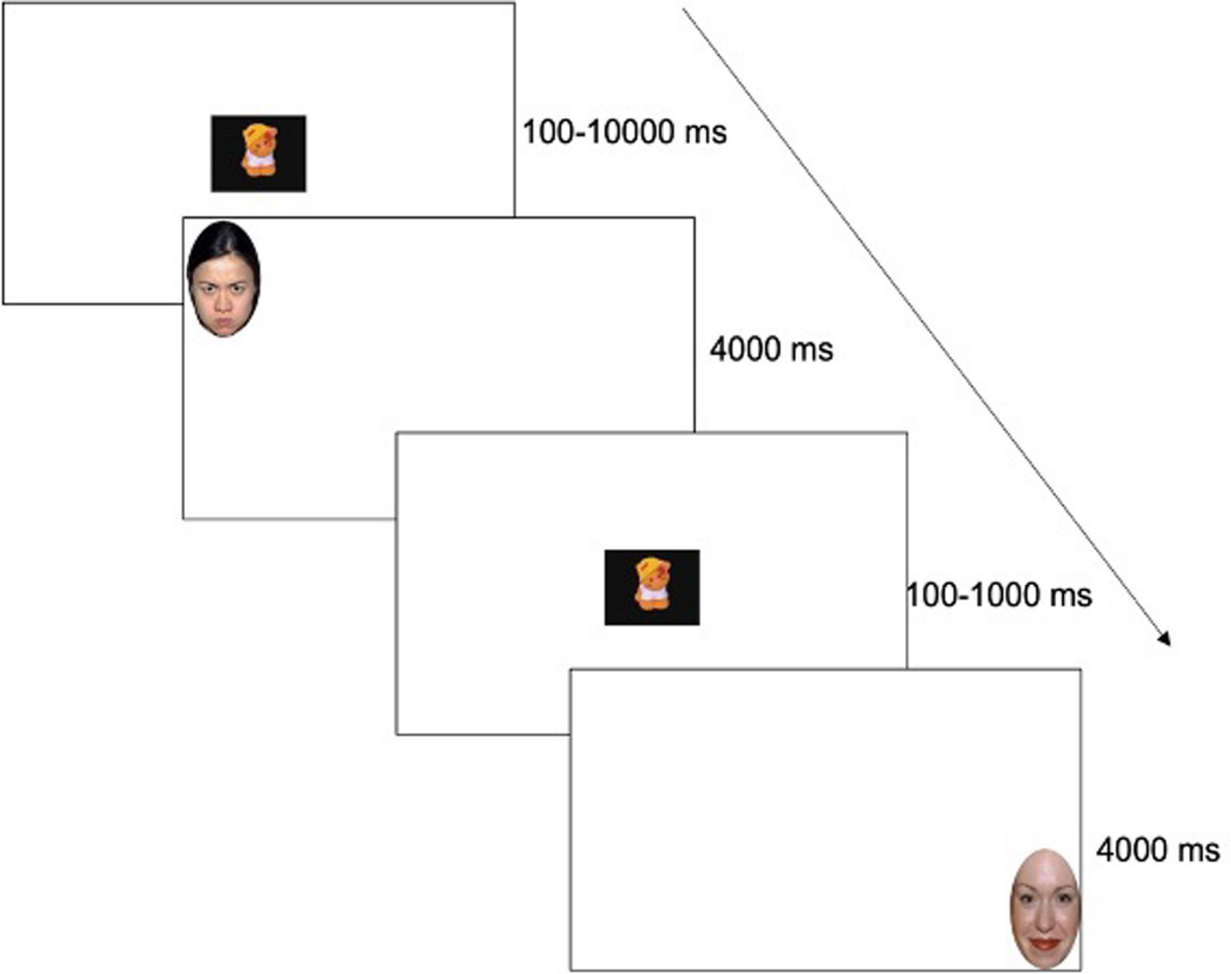
Task schematic for the vigilance task

Eye‐tracking data were collected during an infant vigilance task (Figure 1) to assess infants' ability to detect emotional faces (Fu et al., 2020). The task included 90 trials, and each trial began with a randomly presented fixation‐dependent attention getting video with a black background and classical music dubbed in. Trials were initiated when the infant's attention was on a video clip presented centrally on the screen, which was triggered either when the infant fixated for at least 100‐ms or when the experimenter determined that the infant was looking at the video clip. If the infant did not attend to the center of the screen, the slide advanced after 10,000‐ms. Each trial continued with an emotion facial configuration which randomly appeared in one of the four corners of the screen. Faces were sampled from the NimStim face set (Tottenham et al., 2009) and appeared for up to 4000‐ms or until the participant fixated it for 100‐ms or when the experimenter determined that the infant was looking at the video clip. Ten actors (five men, five women) provided neutral, happy, or angry, closed mouth images. Facial stimuli were approximately 9.50 cm × 6.50 cm and the visual angle of each face was 9.05° (H) × 6.20° (W). Faces were approximately 16.59° visual angle from the center. No face stimuli appeared in the same location consecutively, and the order of face stimuli was randomized across participants. Location of the faces was counterbalanced across the four corners of the screen. There were 4000‐ms white screens that were shown after every seventh trial to minimize habituation and predictive looking. Task design and recording were completed using Experiment Center (SensoMotoric Instruments).

The raw (X,Y) position of fixations were exported from BeGaze (SensoMotoric Instruments). An area of interest (AOI) encircling and including the entire face stimulus was created and exported from BeGaze. A 2‐cm “error margin” was added to each ellipse, to account for deviation permitted in the calibration procedure. Data processing was restricted to gaze data within the face AOI. An in‐house processing script was written in R (R Core Team, 2020) to measure latency to fixate the face AOI on each trial.

#### Data pre‐processing

Metrics were cleaned on a trial‐by‐trial level. Trials in which a fixation was not detected to the face AOI were not included in latency calculations. Additionally, trials with anticipatory eye movements in which latency to fixate the face was less than 200‐ms were removed from the analysis, as research suggests that this is the minimum amount of time required to plan an eye movement (Canfield & Haith, 1991). Average latencies were then calculated for each emotional configuration at each assessment.

#### Missing values and exclusions

Of the 357 infants enrolled in the study, 283 infants provided data on the vigilance task for at least one assessment. Participants were excluded from the analyses if they did not provide any data on the vigilance task for at least one of the five time points (*N* = 74). Participant data points were also excluded if they were outside the acceptable age range for each assessment, defined as the midpoint between each successive assessment (*N* = 12).

Next, we sought to determine the minimum number of trials required for participants to be included in our analyses. Unfortunately, there is no clear standard for how to address this issue in the eye‐tracking literature. Here we sought to establish a data‐driven approach to retain as much data as possible, but eliminate participants whose data were not reliable. We did this by determining the minimum number of trials required to achieve a stable mean latency using two metrics used in a previously reported analysis (described in Burris et al., 2022), that were adapted from Goldsworthy et al. (2016) and Cuypers et al. (2014). This method is based on calculating a rolling mean—or the average latency including all trials up to the current trial—and comparing it to the overall mean latency across all trials. The logic then being to retain only the participants who completed enough trials that their mean latencies could be considered stable.

We first estimated the overall mean latency and its 95% bootstrap confidence interval (CI) for each participant and for each assessment and type of emotion presented. Then, the rolling average latency was computed for each trial (again, the average latency including all trials up to the current trial). For each trial, we recorded (1) the percent difference from the overall mean latency; and (2) whether the rolling average was contained within the 95% CI associated with the overall mean latency. Having done this for all participants, the minimum number of trials necessary to get a stable mean latency was determined as (1) the trial at which the percentage difference from the overall mean latency fell below 10% (on average); and (2) the trial at which the proportion of rolling average latencies that fall into the confidence interval reached 0.95. Ten trials were determined to be the minimum number of trials required to reach both criteria across the assessments and emotions. In other words, before Trial 10, the rolling mean fell outside the 95% CI for the mean across all trials, and thus did not provide a stable estimate of the mean. As a result, data from infants who did not provide at least 10 trials were eliminated.

Based on this criteria, 404 (23%) data points for infants who attempted each task were excluded for having an insufficient number of trials (note 24 of these were also considered outliers, defined as more than 3 standard deviations (*SD*s) from the mean). An additional four (<1%) participant data points were considered outliers, defined as more than 3 *SD*s from the mean for each emotion at each assessment, and were removed. In the end, 1382 participant data points were included in the analyses. Table S1 provides information about the final sample before and after these cleaning metrics, and Figures S1 and S2 display the spread of data before and after cleaning.

### Overlap task

**FIGURE 2:**
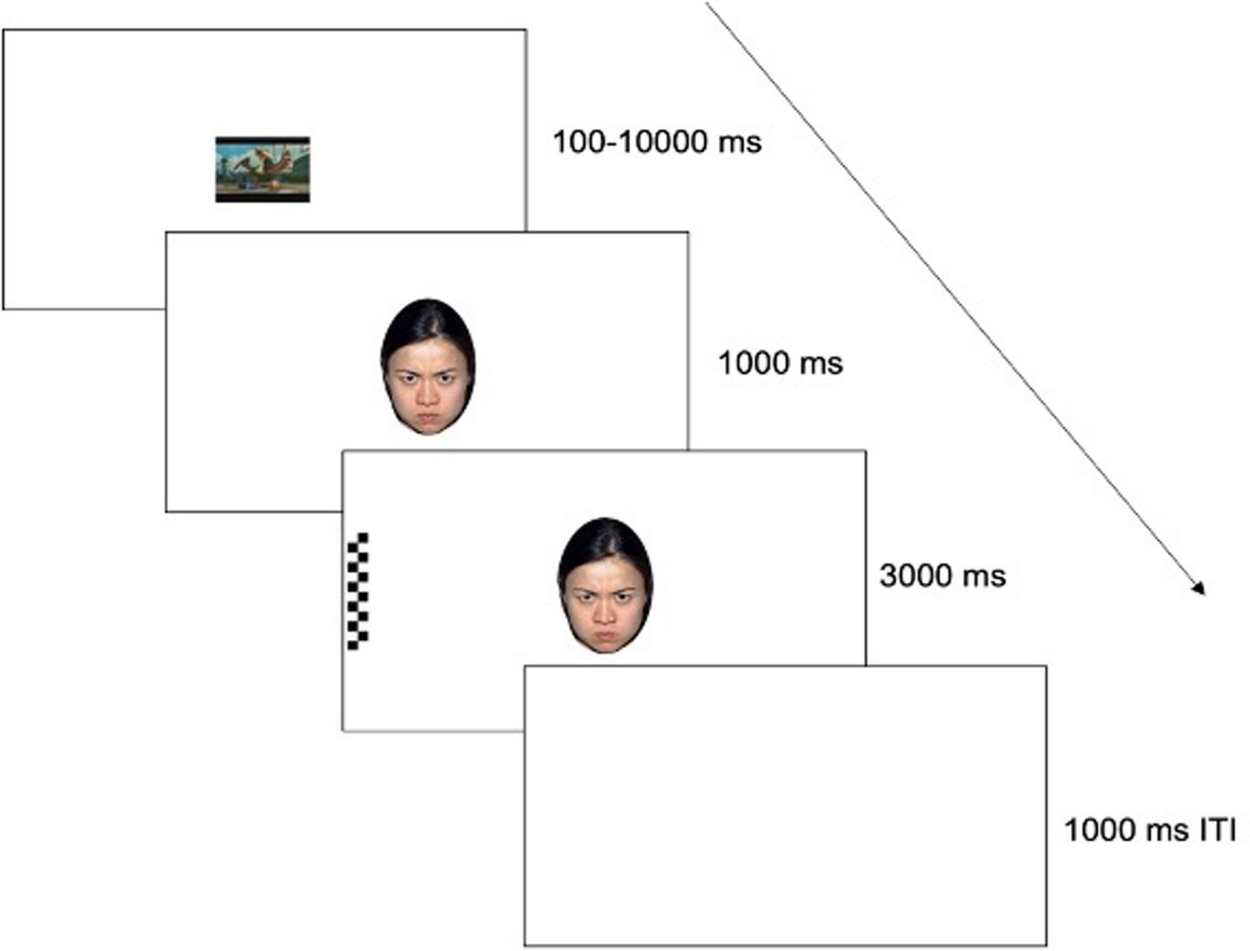
Task schematic for the overlap task

Infants completed a version of a classic overlap task (i.e., Morales et al., 2017; Peltola et al., 2008); originally known as a fixation shift paradigm or gap or overlap paradigm (Atkinson & Braddick, 1985; Colombo, 2001; Hood & Atkinson, 1993; Matsuzawa & Shimojo, 1997), to assess infants' ability to engage with and disengage from emotional facial configurations (Figure 2). Infants were presented with up to 30 experimental trials, ending either when all trials were completed or when the infant could no longer attend to the task. Each trial was initiated when the infant's attention was on a video clip presented centrally on the screen, which was triggered either when the infant fixated for at least 100‐ms or when the experimenter determined that the infant was looking at the video clip. If the infant did not attend to the center of the screen, the slide advanced after 10,000‐ms. Following this was a central face sampled again from the NimStim face set for 1000‐ms (Tottenham et al., 2009). Ten actors (five men) provided neutral, happy, or angry, closed‐mouth images. Facial stimuli were approximately 12 cm × 8 cm and the visual angle of each face was 11.42° (H) × 7.63° (W). Following the presentation of the face, a checkerboard stimulus then appeared in either the left or right periphery of the screen adjacent to the face (20.78° visual angle) for 3000‐ms. The checkerboard was 12‐cm × 2.5‐cm, 11.42° × 2.39° visual angle. This progression of stimuli was concluded with a 1000‐ms intertrial interval, which was a blank screen. No consecutive trials were identical in terms of face and probe placement.

Areas of interest were drawn as ellipses enclosing the face and rectangles enclosing the checkerboards. A 2‐cm “error margin” was added to each ellipse, to account for the deviation permitted in the calibration procedure. Analyses were based on gaze to these designated AOIs. Fixations, defined as gaze maintained for at least 80‐ms within a 100‐pixel maximum dispersion, were extracted with BeGaze (SensoMotoric Instruments). All other computations of gaze metrics were performed using in‐house R scripts.

We examined two outcome measures used in previous research with this task: mean latency to fixate the probe following each emotional facial configuration (disengagement) and mean preferential looking to each emotional face category (engagement with the face) (e.g., Morales et al., 2017; Peltola, Leppänen, Vogel‐Farley, et al., 2009; Vallorani et al., 2021). To obtain the preferential looking scores, for each trial, we first computed total looking time to the central face while the checkerboard stimulus (probe) was present, as well as total looking time to the peripheral probe while the central face was present. We used these values to create a preferential looking score toward each emotion facial configuration, defined as the time looking to each emotion facial configuration divided by the total time looking to the face and probe. We then obtained the mean latency to fixate the probe, and mean preferential looking to the face for each emotion facial configuration at each assessment.

#### Data pre‐processing

Metrics were cleaned on a trial‐by‐trial basis. If a fixation to the checkerboard stimulus was not detected during a trial, the trial was not included in latency calculations. Furthermore, trials with anticipatory eye movements in which latency to fixate the checkerboard probe was less than 200‐ms were also removed from the analysis (Canfield & Haith, 1991). After cleaning, we obtained the mean latency to fixate the face for each emotion category at each assessment. For the preferential looking to the face, trials were included in looking time calculations if gaze was detected toward at least one of the AOIs on the screen when both the face and probe were on the screen during the trial. For each trial, we then calculated a looking preference score to the face, defined as looking time to the face divided by the total looking time to both the probe and the emotion faces. We then obtained a mean for preferential looking to the face for each emotion category at each assessment.

#### Missing values and exclusions

Of the 357 infants enrolled in the study, 279 infants provided data on the overlap task for at least one assessment. Participants were excluded from the analyses if they did not provide any data on the overlap task for at least one of the five assessments (*N* = 78). Participant data points were also excluded if they were outside of the acceptable age range for each assessment, defined by the midpoint between each successive assessment (*N* = 9).

Next, we applied the same technique used on the vigilance task to determine the number of trials infants needed to provide to be included in analyses for each metric in the overlap task. For our measure of disengagement (latency to fixate the probe), infants needed to provide data on at least five usable trials of each emotion category to be included in our analyses. Based on these criteria, we lost a substantial amount of data on this metric, chiefly because infants rarely fixated the probe at all—they only did so on about one‐third of the trials—regardless of age and emotion category. Previous research with young infants has also reported similar patterns of poor data quality and did not report on this metric due to a large number of infants failing to provide even a small number of trials (e.g., Peltola et al., 2013). Given the substantial data loss, we were unable to examine this metric in the remaining analyses using the data that met our inclusion criteria, as the models would not converge. Table S2 presents changes in sample size based on these cleaning metrics, Table S3 presents descriptives after cleaning the data, and Figures S3 and S4 display the spread of data before and after cleaning.

For looking time, our analysis determined that infants needed to provide data on at least six trials for looking to the probe and seven trials for looking to the face. Here, we opted to retain data from infants who provided at least six trials to be included in the calculation of preferential looking to the face. Based on this criteria, 315 (18%) data points for infants who attempted the task were excluded for having an insufficient number of trials (note that 25 [1%] data points were also considered outliers, defined as more than 3 *SD*s from the mean for each emotion at each assessment). In the end, 1416 participant data points were included in the preferential looking to the face analyses. Table S2 presents changes in sample based on these cleaning metrics, and Figures S5 and S6 display the spread of data before and after cleaning.

### Data analytic strategy

All statistical analyses were conducted using R version 4.0.3 (R Core Team, 2020). For each task, we first provide descriptives for all outcome metrics. Following this, we examined infant trajectories of attention related to emotional facial configurations over time using multilevel growth curve modeling using the lme4 package in R (Bates et al., 2015). Restricted maximum likelihood (REML) estimation was used to provide unbiased statistical estimates of model parameters with missing data (Enders, 2010). Maximum likelihood (ML) estimation was used when comparing model fit. For all models, we compare trajectories of angry to neutral and happy to neutral facial configurations. Given that both tasks relied on a nested structure of metrics nested within emotion configuration categories, the final model selections were determined based on both study design and model comparison. We first present data from the vigilance task, which taps into the alerting network and measures vigilance toward angry, happy, and neutral facial configurations. We then present data on the overlap task, which taps into the orienting network and measures engagement with and disengagement from the same three emotion categories.

## RESULTS

### Vigilance

#### Descriptive statistics

Descriptive statistics of infants' performance on the vigilance task are presented in Table 2. Table 3 provides the zero‐order correlations on all vigilance metrics for each emotion configuration at each assessment. In terms of data loss, a lower proportion of data was retained after cleaning based on the minimum number of trials and removal of outliers at the earlier assessments (about 64% at 4 months) compared to later assessments (about 83% at 24 months, see Table S1). Infants completed a similar number of trials for each emotional facial configuration at each assessment, and we observed a general improvement in the number of trials completed between 4 months (46% of trials) and 8 months of age (54% of trials), with infants completing 64% of trials on average by 24 months of age.

**TABLE 2:** Descriptive statistics for emotion facial configurations in the vigilance task

	Assessment	Mean latency (ms)						Number of trials				
		*n*	Mean	*SD*	*SE*	Min	Max	Mean	*SD*	*SE*	Min	Max
Neutral facial configuration	4M	62	638.47	210.07	26.68	344.65	1371.14	19.27	5.55	0.70	10	29
	8M	141	499.66	143.47	12.08	300.01	1158.36	19.48	5.25	0.44	10	29
	12M	108	493.74	125.37	12.06	319.57	901.53	20.85	5.00	0.48	10	30
	18M	92	501.63	157.79	16.45	317.31	1114.52	20.85	5.42	0.57	10	30
	24M	55	529.07	160.60	21.66	306.01	998.35	21.24	5.70	0.77	10	30
Angry facial configuration	4M	61	666.17	213.37	27.32	405.56	1336.39	19.15	5.68	0.73	10	30
	8M	142	508.98	146.75	12.32	322.23	1086.69	19.87	5.11	0.43	10	29
	12M	110	508.11	135.57	12.93	313.70	946.69	21.37	5.50	0.52	10	30
	18M	94	492.53	135.26	13.95	313.90	946.45	21.36	5.46	0.56	10	30
	24M	52	478.19	114.71	15.91	326.20	814.12	22.83	5.17	0.72	10	30
Happy facial configuration	4M	63	647.98	197.71	24.91	351.05	1257.17	18.63	5.63	0.71	10	30
	8M	145	519.70	156.27	12.98	305.36	1100.02	19.52	5.13	0.43	10	28
	12M	110	480.13	115.63	11.02	320.15	923.93	20.57	5.49	0.52	10	29
	18M	93	521.24	140.09	14.53	331.04	1016.69	21.37	4.80	0.50	10	30
	24M	54	523.52	162.24	22.08	340.48	1134.87	21.70	5.54	0.75	11	30

*Note*: M, months.

**TABLE 3:** Zero‐order correlations for the vigilance task

	4M Neu	4M Ang	4M Hap	8M Neu	8M Ang	8M Hap	12M Neu	12M Ang	12M Hap	18M Neu	18M Ang	18M Hap	24M Neu	24M Ang	24M Hap
4M Neu															
4M Ang	**.648**														
4M Hap	**.591**	**.549**													
8M Neu	−.116	−.084	−.168												
8M Ang	−.032	−.017	−.084	**.449**											
8M Hap	−.019	−.086	−.082	**.541**	**.644**										
12M Neu	.330	−.126	.111	**.290**	.084	.177									
12M Ang	.073	.004	.073	.103	.115	.048	**.528**								
12M Hap	.276	.180	.206	−.044	−.095	−.146	**.550**	**.479**							
18M Neu	.189	.267	.063	−.064	.005	.085	.119	.171	.251						
18M Ang	.195	−.336	.143	**.469**	−.128	.069	**.328**	.144	.183	**.612**					
18M Hap	−.157	−.288	.137	.313	.092	.265	.150	.106	.135	**.558**	**.576**				
24M Neu	−.235	.208	−.439	−.079	.080	−.111	.306	.037	**.425**	−.045	.153	.107			
24M Ang	−.240	−.098	−.307	.068	−.170	−.266	.349	.222	**.398**	−.020	.053	.060	**.615**		
24M Hap	−.215	−.013	−.387	−.181	**.513**	.041	.206	.010	.088	.010	.068	.103	**.641**	**.542**	

*Note*: M, months; Neu, neutral facial configuration; Ang, angry facial configuration; hap, happy facial configuration. Significant correlations at *p* < .05 are denoted in bold type.

#### Model selection

**FIGURE 3:**
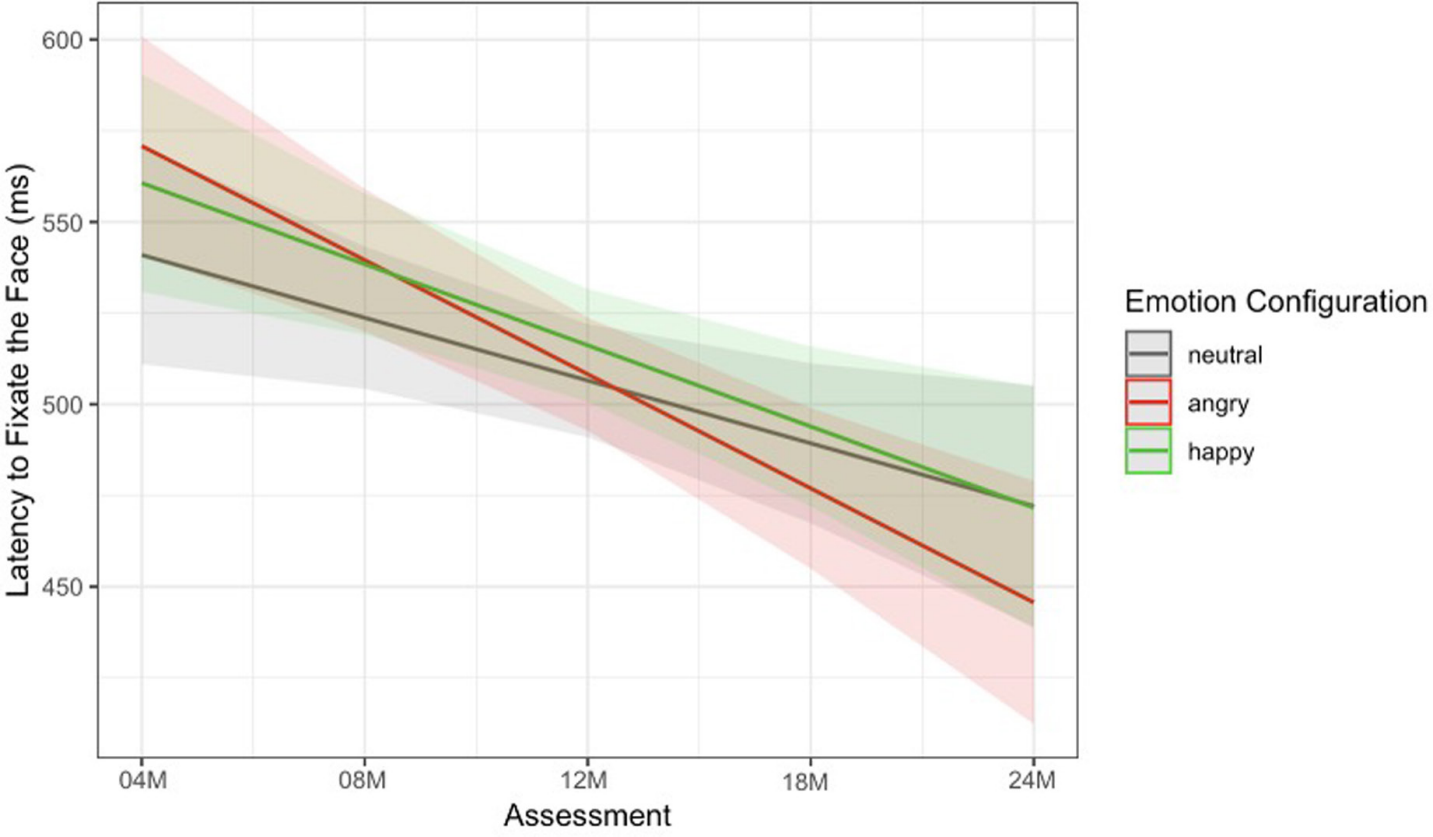
Final model predictions: Latency to fixate the face (vigilance task)

We used multilevel linear growth modeling to measure latency and the longitudinal change in latency to fixate each emotional facial configuration over time. In the following analyses, we were most interested in whether infants' attention to emotional facial configurations differed by emotion category over time, and compared trajectories of angry and happy facial configurations to neutral facial configurations as the reference. The final model was determined based on both study design (due to the multilevel nature of latencies nested within emotion facial categories) and model comparison and is presented in the text and in Figure 3. Additional information about model selection is provided in the Supplementary Analysis code.

The final model included a random intercept, age as a fixed and random effect, and emotion as a fixed effect. The final model showed significant change over time in relative attention to emotional stimuli (*b* = −17.21, *p* = .015, 95% CI [−30.962, −3.461]), with infants becoming faster to fixate all facial configurations as they got older. Furthermore, the model showed a significant difference in attention to angry relative to neutral facial configurations (*b* = 29.89, *p* = .043, 95% CI [−27.067, −1.097]), as well as a significant difference in rate of change in attention for rapid detection to angry relative to neutral facial configurations over time (*b* = −14.08, *p* = .034, 95% CI [−27.07, −1.10]). As you can see in Figure 3, as infants got older, latency to fixate angry facial configurations accelerated faster compared to neutral configurations. There were no significant differences in attention to happy facial configurations relative to neutral configurations, nor in the rate of change in attention to happy and neutral facial configurations over time.

Given that we only found a significant effect of time for angry compared to neutral facial configurations, we then conducted paired samples *t*‐tests at each assessment to examine whether fixations to angry facial configurations were faster than neutral ones, indicative of an attention bias to threat. Our results show that infants were only faster to fixate angry (*M* = 478.19, *SD* = 114.71) versus neutral facial configurations (*M* = 529.07, *SD* = 160.60) at 24 months of age, *t*(51) = 2.22, *p* = .031, 95% CI [3.449, 69.452], with no significant differences emerging prior (*p*'s > .337), suggesting that a significant bias for the rapid detection of threat did not emerge until around 24 months of age.

### Overlap

#### Descriptive statistics

Descriptive statistics of infants' preferential looking to the face on the overlap task and zero‐order correlations are presented in Tables 4 and 5. We retained at least 74% of the data at each assessment in the final data, and infants completed approximately eight trials on average across all emotion categories and ages for this metric.

**TABLE 4:** Descriptive statistics for the overlap task (preferential looking to the face)

	Assessment	Mean preferential looking to the face						Number of trials				
		*n*	Mean	*SD*	*SE*	Min	Max	Mean	*SD*	*SE*	Min	Max
Neutral facial configuration	4M	84	0.88	0.13	0.01	0.44	1.00	8.82	1.30	0.14	6	10
	8M	144	0.89	0.08	0.01	0.54	1.00	8.53	1.44	0.12	6	10
	12M	95	0.90	0.08	0.01	0.68	1.00	8.42	1.36	0.14	6	10
	18M	95	0.89	0.10	0.01	0.60	1.00	8.80	1.25	0.13	6	10
	24M	54	0.85	0.12	0.02	0.58	1.00	8.44	1.42	0.19	6	10
Angry facial configuration	4M	80	0.87	0.14	0.02	0.48	1.00	8.81	1.37	0.15	6	10
	8M	139	0.88	0.09	0.01	0.59	1.00	8.61	1.46	0.12	6	10
	12M	101	0.90	0.09	0.01	0.57	1.00	8.42	1.38	0.14	6	10
	18M	95	0.90	0.08	0.01	0.62	1.00	8.93	1.29	0.13	6	10
	24M	55	0.87	0.11	0.02	0.60	1.00	8.82	1.17	0.16	6	10
Happy facial configuration	4M	81	0.89	0.13	0.01	0.53	1.00	8.86	1.38	0.15	6	10
	8M	143	0.90	0.08	0.01	0.61	1.00	8.64	1.33	0.11	6	10
	12M	100	0.90	0.08	0.01	0.68	1.00	8.46	1.37	0.14	6	10
	18M	95	0.90	0.11	0.01	0.51	1.00	8.88	1.28	0.13	6	10
	24M	55	0.86	0.13	0.01	0.51	1.00	8.50	1.37	0.19	6	10

*Note*: M, months.

**TABLE 5:** Zero‐order correlations for the overlap task (preferential looking to the face)

	4M Neu	4M Ang	4M Hap	8M Neu	8M Ang	8M Hap	12M Neu	12M Ang	12M Hap	18M Neu	18M Ang	18M Hap	24M Neu	24M Ang	24M Hap
4M Neu															
4M Ang	.**761**														
4M Hap	**.762**	**.868**													
8M Neu	.288	.228	.288												
8M Ang	.070	.115	.063	**.497**											
8M Hap	.046	.057	−.050	**.655**	**.571**										
12M Neu	.005	.057	.144	.196	.170	.178									
12M Ang	.053	−.023	.131	**.308**	.110	**.339**	**.452**								
12M Hap	.074	.143	.100	**.396**	.078	.167	**.480**	**.448**							
18M Neu	**−.438**	**−.448**	−.199	.054	.106	.017	.104	.218	.193						
18M Ang	−.299	−.296	−.055	.096	.021	.084	.140	.157	.282	**.744**					
18M Hap	−.416	−.346	−.155	−.024	.085	−.024	.006	.238	.199	**.683**	**.549**				
24M Neu	−.533	−.601	−.329	−.184	−.164	.064	.143	.379	−.162	**.478**	**.368**	**.428**			
24M Ang	−.484	−.412	−.059	.151	−.019	.096	.107	.357	−.084	**.328**	.250	.197	**.723**		
24M Hap	−.392	**−.594**	−.320	−.118	−.088	.155	.318	.263	−.115	**.482**	**.436**	**.333**	**.740**	**.697**	

*Note*: M, months; Neu, neutral facial configuration; Ang, angry facial configuration; Hap, happy facial configuration. Significant correlations at *p* < .05 are denoted in bold type.

#### Model selection

**FIGURE 4:**
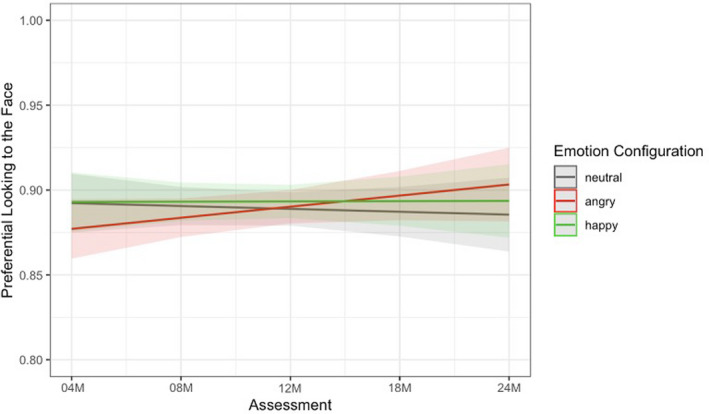
Final model predictions: Engagement with the face (overlap task)

For the overlap task, we used multilevel linear growth modeling to measure preferential looking to the face and the longitudinal change in preference to each emotional facial configuration relative to the probe over time. Again, preferential looking to the face was defined as the amount of time infants looked at the face divided by the total looking time to the face and the probe when both stimuli were presented on the screen for each emotional facial configuration (angry, happy, neutral). Given the literature on infants' preference for faces early in life (e.g., Johnson et al., 1991), we expected that infants would display a consistently high preference for faces across time. We examined whether infant's attention to angry and happy facial configurations differed from neutral facial configurations over time. The final model was determined based on both study design (due to the multilevel nature of looking time nested within emotional facial categories) and model comparison and is presented in the text and in Figure 4. Additional information about model selection is presented in the Supplementary Analysis code.

The final model included a random intercept, age as a fixed and random effect, and emotion as a fixed effect. The final model (Figure 4) shows a nonsignificant difference in preferential looking to angry relative to neutral facial configurations (*b* = −0.002, *p* = .057, 95% CI [−0.031, 0.000]), and a significant difference in the rate of change in attention for preferential looking to angry relative to neutral facial configurations over time (*b* = 0.008, *p* = .024, 95% CI [0.001, 0.015]), though this difference is small. As you can see in Figure 4, infants' preferential looking to angry facial configurations increased more compared to neutral configurations. There were no significant differences between preferential looking to the face for happy and neutral facial configurations.

We then conducted paired samples *t*‐tests at each assessment to examine whether infants displayed a greater preference for angry facial configurations than neutral ones, indicative of an attention bias to threat. Our results show that infants displayed a stronger preference for angry (*M* = 0.87, *SD* = 0.11) versus neutral facial configurations (*M* = 0.85, *SD* = 0.12) at 24 months of age, *t*(50) = −2.22, *p* = .031, 95% CI [−0.052, −0.003], with no significant differences emerging prior (*p'*s > .210), suggesting that a significant bias for engagement with threat does not emerge until around 24 months of age.

## DISCUSSION

The present study examined patterns of attention toward emotional stimuli (angry, happy, and neutral facial configurations), with a specific focus on threat processing across the first 2 years of life in a longitudinal sample of infants using two eye‐tracking tasks that were strategically designed to tap into separate components of attention. When we examined alerting via the vigilance task, we found that infants became faster to detect all emotional facial configurations over time. This is in line with previous research demonstrating that saccade reaction times and accuracy generally improve with age, with infants becoming faster and more accurate, respectively (e.g., Alahyane et al., 2016). Furthermore, between 4 and 24 months of age, infants became faster to detect angry relative to neutral facial configurations. This difference was not significant for happy versus neutral faces. Moreover, we found that significant differences in infants' rapid detection of angry relative to neutral facial configurations were not present until 24 months of age.

We found similar looking patterns over time in orienting, as indexed by the overlap task. This is consistent with previous research suggesting that infants who tend to fixate emotional facial configurations particularly quickly also tend to look longer at those same images (Vallorani et al., 2021). Overall, infants displayed a strong preference for looking at any facial configuration over the checkerboard probe, with infants spending approximately 70% of their time looking at the face when the probe was also present on the screen. In fact, based on the raw data, infants only shifted their attention to the probe on an average of 3.70 of 10 trials for each emotion category across assessments. Importantly, infants developed a stronger preference for looking at angry over neutral configurations relative to looking at the probe over time. No such change was found when comparing happy to neutral configurations. Furthermore, we found that infants displayed a significant bias for threat in engagement at 24 months, showing a greater preference for angry over neutral facial configurations in the presence of a probe.

Altogether, this study documents the emergence of attention biases for threat longitudinally over the first 2 years of life. Data from both eye tracking tasks suggest that between 4 and 24 months, infants became faster to detect angry facial configurations over time, and demonstrated greater engagement with threatening facial configurations (longer looking toward angry faces) over time relative to neutral configurations. These patterns were not present when we examined trajectories of happy relative to neutral facial configurations. Furthermore, across both measures of attention, a clear bias for angry facial configurations was present only at 24 months of age in our sample.

Our findings suggest that while biased attention to threat might be normative, this pattern is undergoing significant developmental change across the first 2 years of life. Specifically, we found that an attention bias for angry facial configurations was not evident before 24 months of age in our sample of infants. Moreover, while the goal of this paper was not to test competing models, but rather to describe trajectories of attention toward affective stimuli, the current findings do not provide support for the integral bias model proposed by Field and Lester (2010). Of the three models proposed by Field and Lester (2010), our data provide some support for the acquisition model, such that a bias for threat is not present from birth but emerges over time. Here, we demonstrated that affective attention develops normatively over the course of the first 2 years of life, with a preference for angry facial configurations not emerging until the end of the second year of life.

One question that remains is what is driving this developmental change in attention to threat, and why do we see a shift in the prioritization of threat by 24 months of age? One possibility is that we are capturing a novelty effect, such that once infants can discriminate between different emotional facial configurations, they become interested in angry configurations because of their novelty and unfamiliarity. However, we think that this explanation is unlikely, given that infants begin to show a significant looking time preference for fearful facial configurations in a similar overlap task by 7 months of age (e.g., Peltola et al., 2008; Peltola, Leppänen, Mäki, et al., 2009). Here, such a preference for angry faces did not appear until 24 months**—**much later in development. This result suggests that novelty (which has been used to explain the bias for fear at 7 months) may not explain why a bias for anger only emerges after the first 2 years of life.

An alternative possibility is that infants begin to show a bias for anger when they begin to associate various emotional facial configurations with predictable behavioral outcomes. For example, between 10 and 14 months of age, infants associate angry facial configurations with frustrating events (Ruba et al., 2020), and between 12 and 18 months of age, infants begin to use negative emotional facial configurations to guide their own actions (Mumme et al., 1996; Mumme & Fernald, 2003; Sorce et al., 1985; Tamis‐Lemonda et al., 2008). Thus, infants may begin to prioritize certain emotions in multiple components of visual attention, including orienting, engagement, and disengagement, around the same time or after they begin to use emotional facial expressions to predict others' actions and plan their own behaviors.

Previous studies examining just a single time point or using just a single task have attempted to pinpoint the presence or absence of these biases at particular ages. However, the current work is suggestive of a continuous and coherent developmental pattern across different attention components, with faster detection and greater engagement with angry facial configurations by 24 months of age. This is the first study to show that patterns of attention related to angry facial configurations in rapid detection and engagement might follow a similar developmental trajectory, undergoing significant developmental change in the same direction between 4 and 24 months. In terms of the broader literature, while the current study found complementary results across different components of attention, it remains unclear whether the results are specific to the types of stimuli used in the current study (i.e., angry facial configurations) or whether the results are specific to this sample. In the current study, we only compared trajectories of attention bias to angry facial configurations to neutral faces. However, it is possible that the trajectories we found are not specific to threat, but instead apply to negative emotions more generally (e.g., angry, fear, sad). We think this is unlikely, given that previous research (e.g., LoBue, 2009) has shown different patterns for looking at emotional configurations that were negative (e.g., sad faces) compared to negative emotional configurations that were also threatening (e.g., anger, fear), but future work is needed to further address this issue. An alternative possibility is that the trajectories we found here are specific to angry facial configurations, or direct signals of threat. It is possible that trajectories would differ for fearful facial configurations, which are threatening, but indirect, indicating that threat is somewhere in the environment. Again, future work is needed to address this issue.

Although this study has several strengths, we must also acknowledge its limitations. First, while these tasks have been used with young infants in previous research (e.g., Fu et al., 2020; Morales et al., 2017), we cannot completely discount the possibility that the lack of a bias before 24 months of age is driven by measurement error, such that younger infants may have struggled with meeting the task demands more than the older participants (e.g., sitting in front of and staring at a bright screen for an extended period of time). However, we think this is unlikely as we did not see evidence of systematic improvement of usable data with age across the tasks.

Second, we were unable to examine infants' latency to fixate the checkerboard probe following each emotional facial configuration in the overlap task after using our data‐driven approach to inclusion and exclusion criteria. After removing participants with an insufficient number of trials and outliers, we retained approximately one‐third of the data across all emotion categories and assessments. In fact, infants rarely fixated the probe at all**—**they only did so on about one‐third of the trials**—**regardless of age and emotion category.

This is not necessarily surprising. Previous studies have also reported significant data loss using this metric (e.g., Morales et al., 2017; Peltola et al., 2013). Furthermore, facial stimuli are incredibly compelling for infants, and it is likely that the design of this task was such that the probe was not compelling enough to encourage disengagement from the facial stimuli for substantial periods of time. Indeed, infants demonstrate a strong preference for faces over other stimuli early in life (e.g., Valenza et al., 1996), and faces convey more information than a static black and white checkerboard probe. In line with previous versions of this task (e.g., Peltola et al., 2008; Peltola, Leppänen, Vogel‐Farley, et al., 2009), the face was also presented centrally on the screen prior to the presentation of the probe and was larger and arguably more interesting than the peripheral checkerboard. This feature of the task could be viewed as a strength, as infants who do quickly disengage from faces or specific emotional facial configurations might show specific patterns of emotional behavior**—**an issue that can be explored in future research.

One final limitation of this work is that our results cannot directly speak to the mechanism for why infants show different patterns of attention for different emotion facial configurations. It is possible that infants' responses are based on the meaning of the emotional facial configurations, as discussed above, but an alternative possibility is that low‐level features of the stimuli were driving our results. Previous research has demonstrated, for example, that perceptual features of various emotional facial configurations, such as the V‐shaped eyebrow associated with stereotypically angry facial configurations are detected faster than inverted “V” shapes (LoBue & Larson, 2010; Tipples et al., 2002). Future longitudinal studies examining different components of attention using more varied emotional facial configurations are needed to further speak to this issue.

Taken together, the current study is one of the first to document affect‐biased attention across the first 2 years of life in a longitudinal sample of infants on two different components of the attention network**—**alerting and orienting. In doing so, we found that infants display differential patterns of attention toward angry facial stimuli, with a clear bias for threat emerging by 24 months of age across several components of attention. By establishing the typical developmental trajectory of attention biases for threat, future research can begin to determine how both individual characteristics of the child (e.g., biology, temperament) and the environment (e.g., parenting, community) impact trajectories of vigilance, engagement with, and disengagement from threat, when normative trajectories of attention bias diverge, and how these divergent trajectories might confer risk for anxiety later in life.

## FUNDING INFORMATION

The study was supported by a grant from the National Institute of Mental Health to Drs. Koraly Pérez‐Edgar, Kristin Buss, and Vanessa LoBue (R01MH109692).

## Supporting information


Appendix S1

Appendix S2

Appendix S3
