# Supplementary material for: Developmental patterns of affective attention across the first 2 years of life
Source: Child Dev. 2022 Jul 29;93(6):e607–21. doi: 10.1111/cdev.13831 (PMC9796239; doi:10.1111/cdev.13831)

**Supplement**

*Table S1: Data Loss for the Vigilance Task*


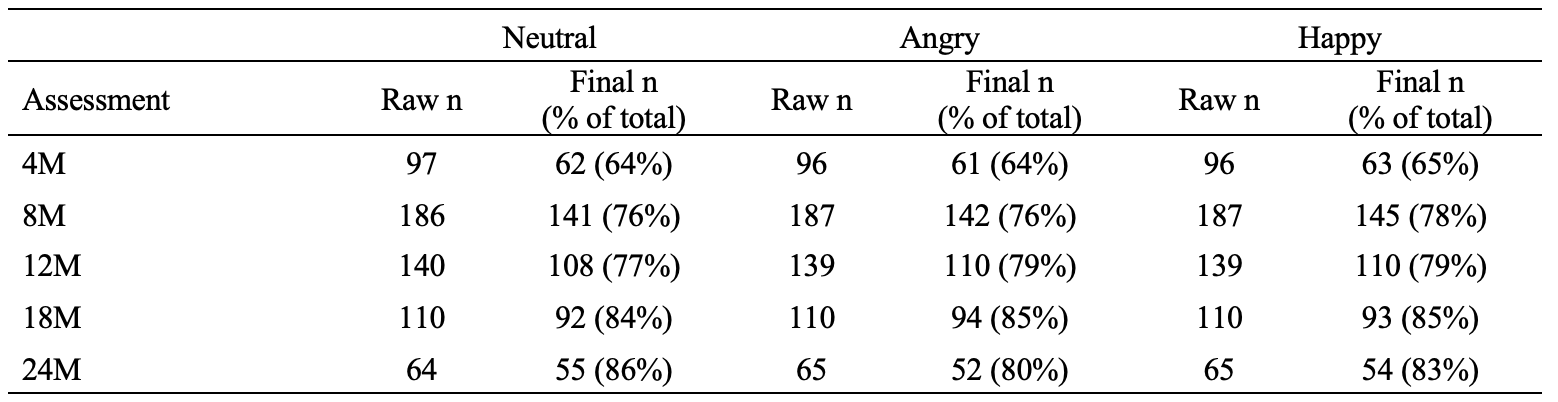


*Note.* For latency to fixate each emotion facial configuration, 404 data points across assessments and emotion facial configurations were dropped due to having an insufficient number of trials (of these, 25 were outliers). An additional 4 data points were dropped based on being an outlier, defined as > 3SD from the mean from each assessment and emotion configuration.

*Table S2: Data Loss for the Overlap Task*

*
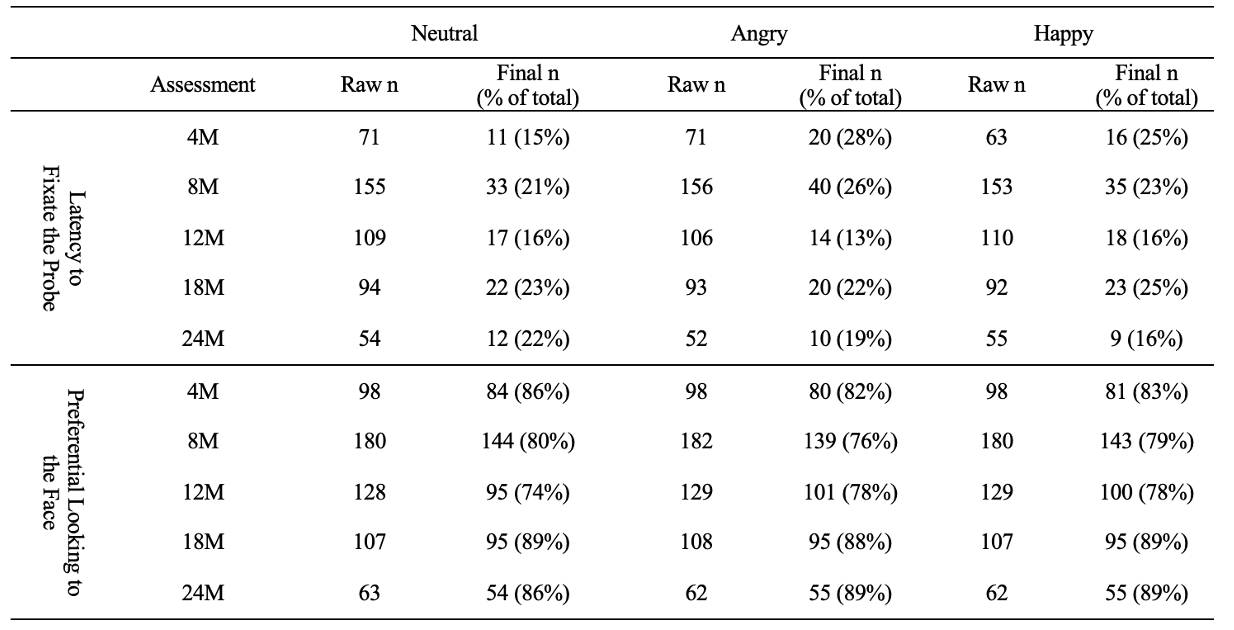
*

*Note.* It is noteworthy that we saw a substantial loss of data for latency to fixate the probe based on these criteria. Specifically, of the remaining data, 1134 (79%) of the 1435 data points across the 5 timepoints and 3 emotion categories did not meet inclusion criteria and were excluded from the final analyses. This included 1104 (77%) data points for participants who attempted each task were excluded for having an insufficient number of trials (30 data points were also considered outliers, defined as more than 3 standard deviations from the mean for each emotion at each timepoint). After this cleaning, we retained about 23% of the data at 4-months, and only 19% of the data by 24-months. When we examined the number of trials infants completed, we saw that infants completed about 6 of 10 trials for this measure (from the raw data, infants only looked at the probe on 3/10 trials on average). For preferential looking to each emotion facial configuration, 315 (18%) of the 1731 data points across assessments and emotion facial configurations were dropped due to having an insufficient number of trials (of these, 25 were outliers, defined as > 3SD from the mean from each assessment and emotion configuration).

*Table S3.* Descriptive Statistics for Emotion Facial Configurations in the Overlap Task (Latency to Fixate the Probe)


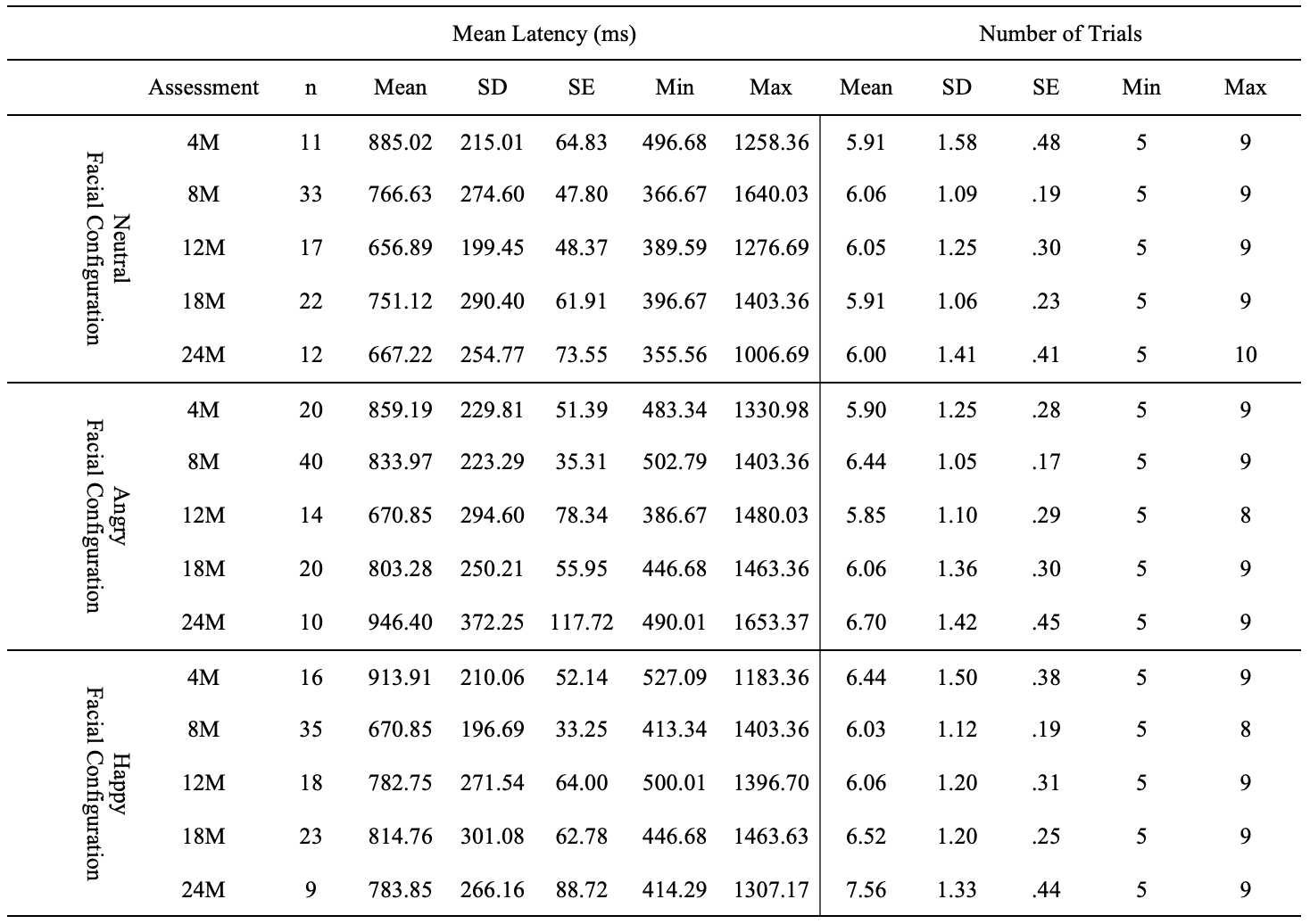


*Figure S1. Spread of Raw Data from the Vigilance Task (before cleaning)*


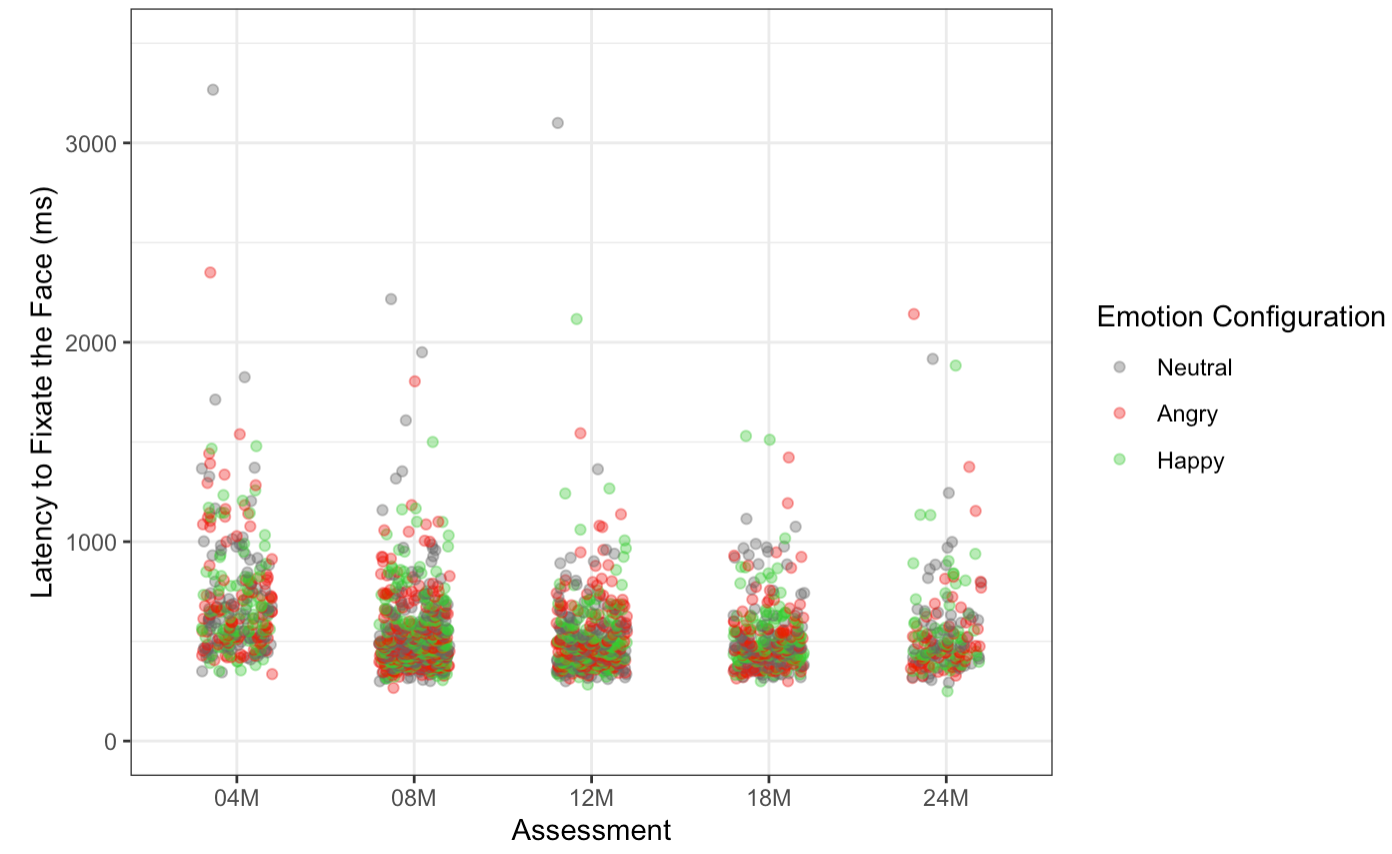


*Figure S2. Spread of Final Data from the Vigilance Task (after cleaning for outliers and insufficient trials)*


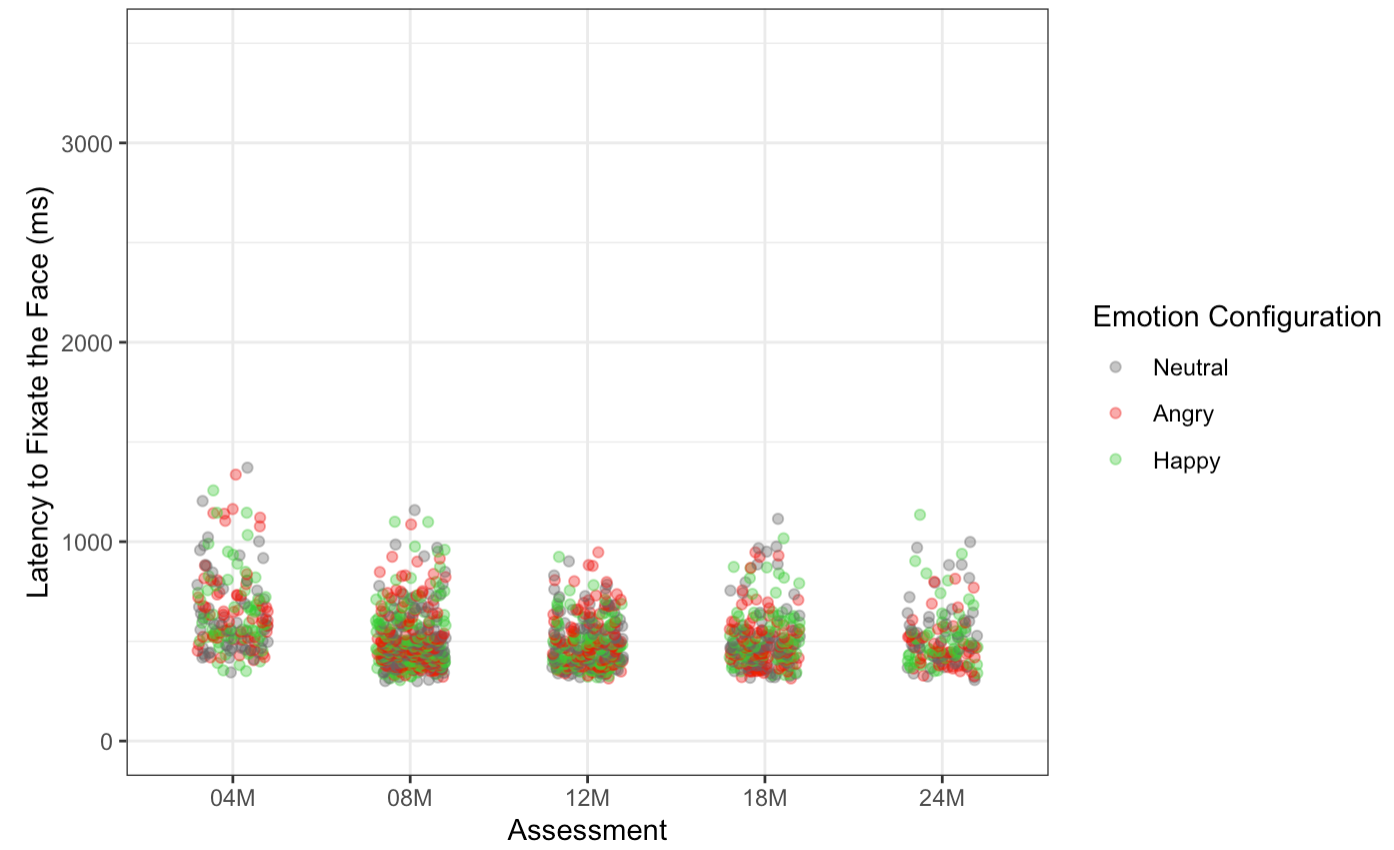


*Figure S3. Spread of Raw Data from the Overlap Task- Latency to Fixate the Probe (before cleaning)*


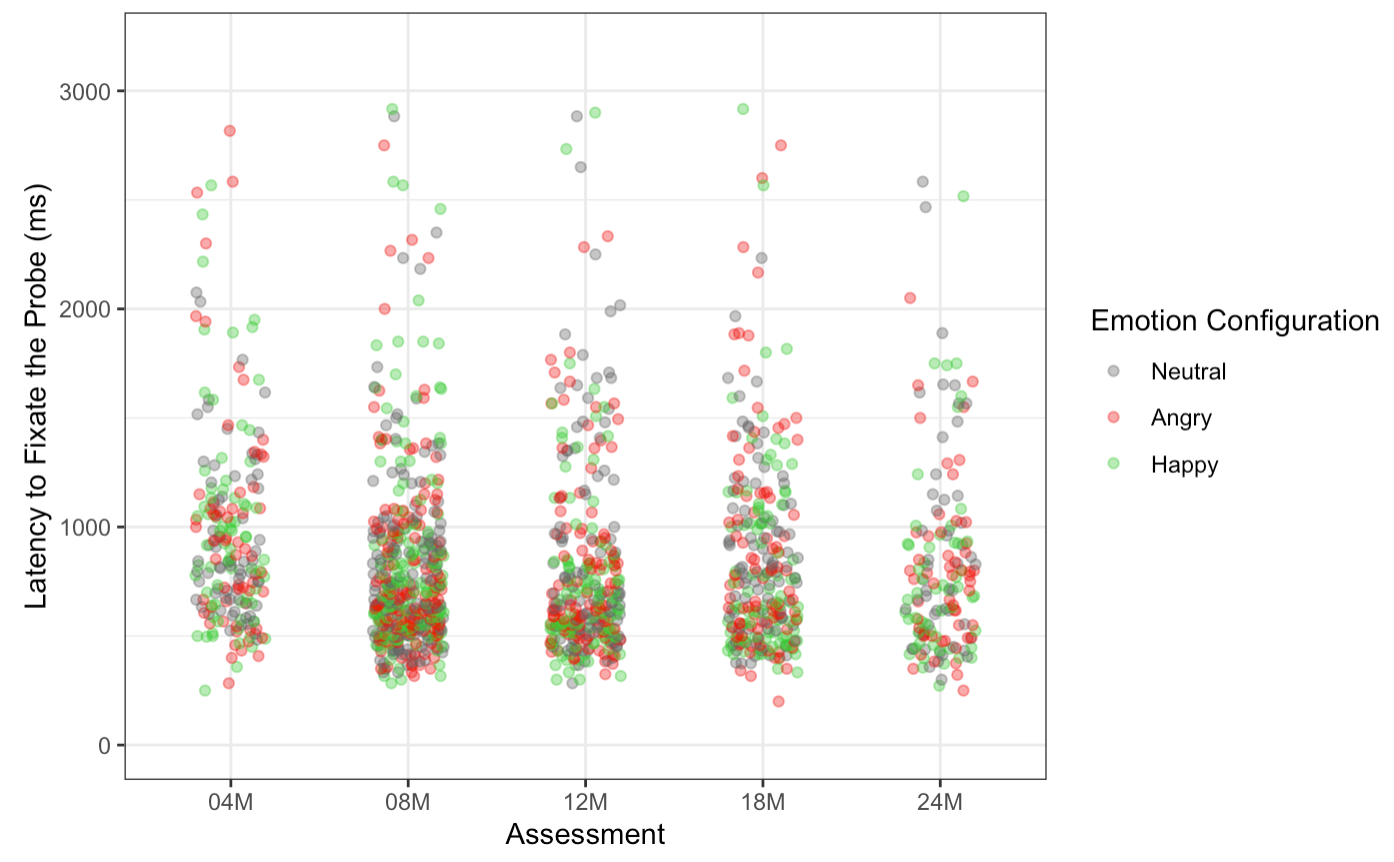


*Figure S4. Spread of Final Data from the Overlap Task, Latency to Fixate the Probe (after cleaning for outliers and insufficient trials)*


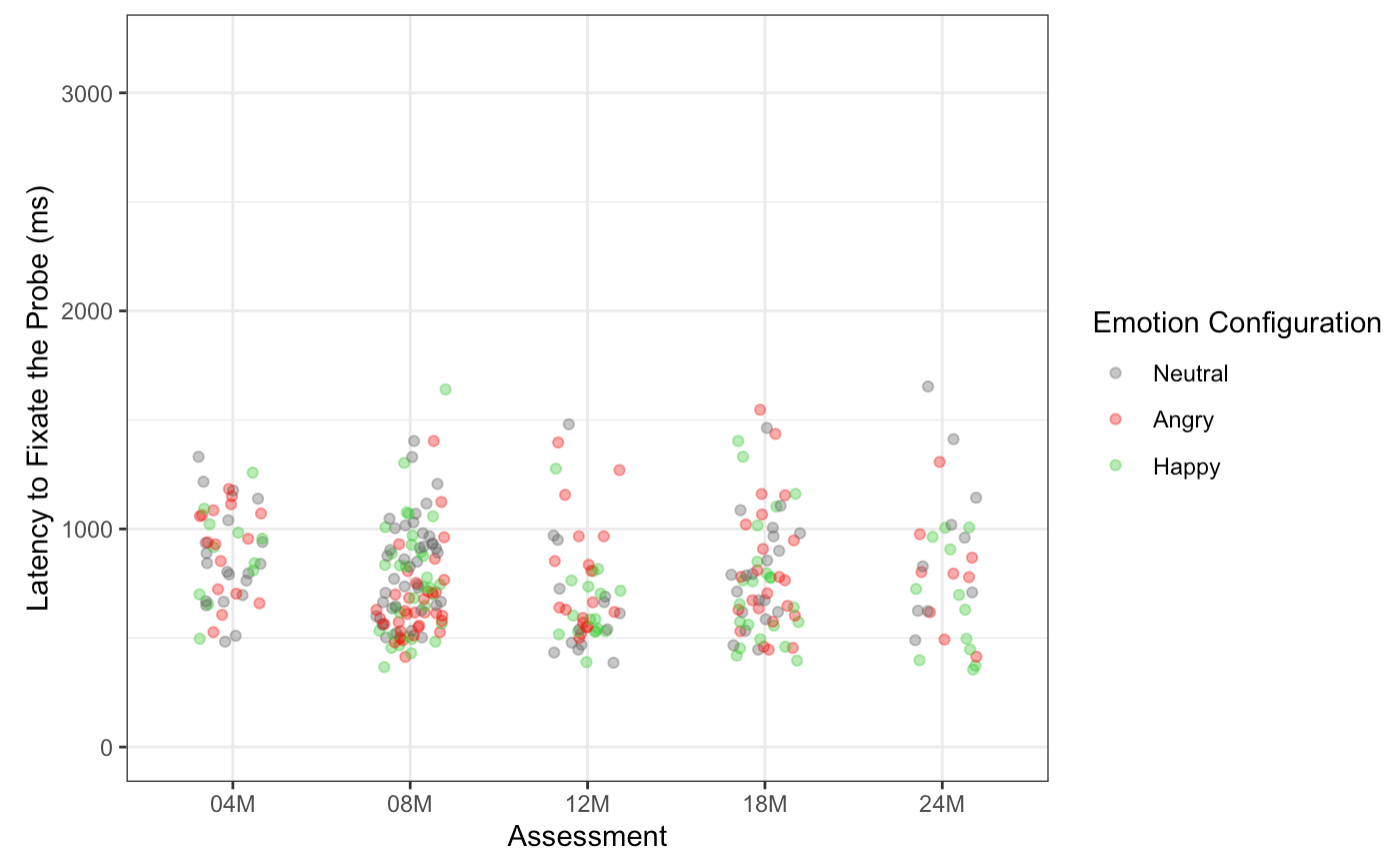


*Figure S5. Spread of Raw Data from the Overlap Task- Preferential Looking to the Face (before cleaning)*


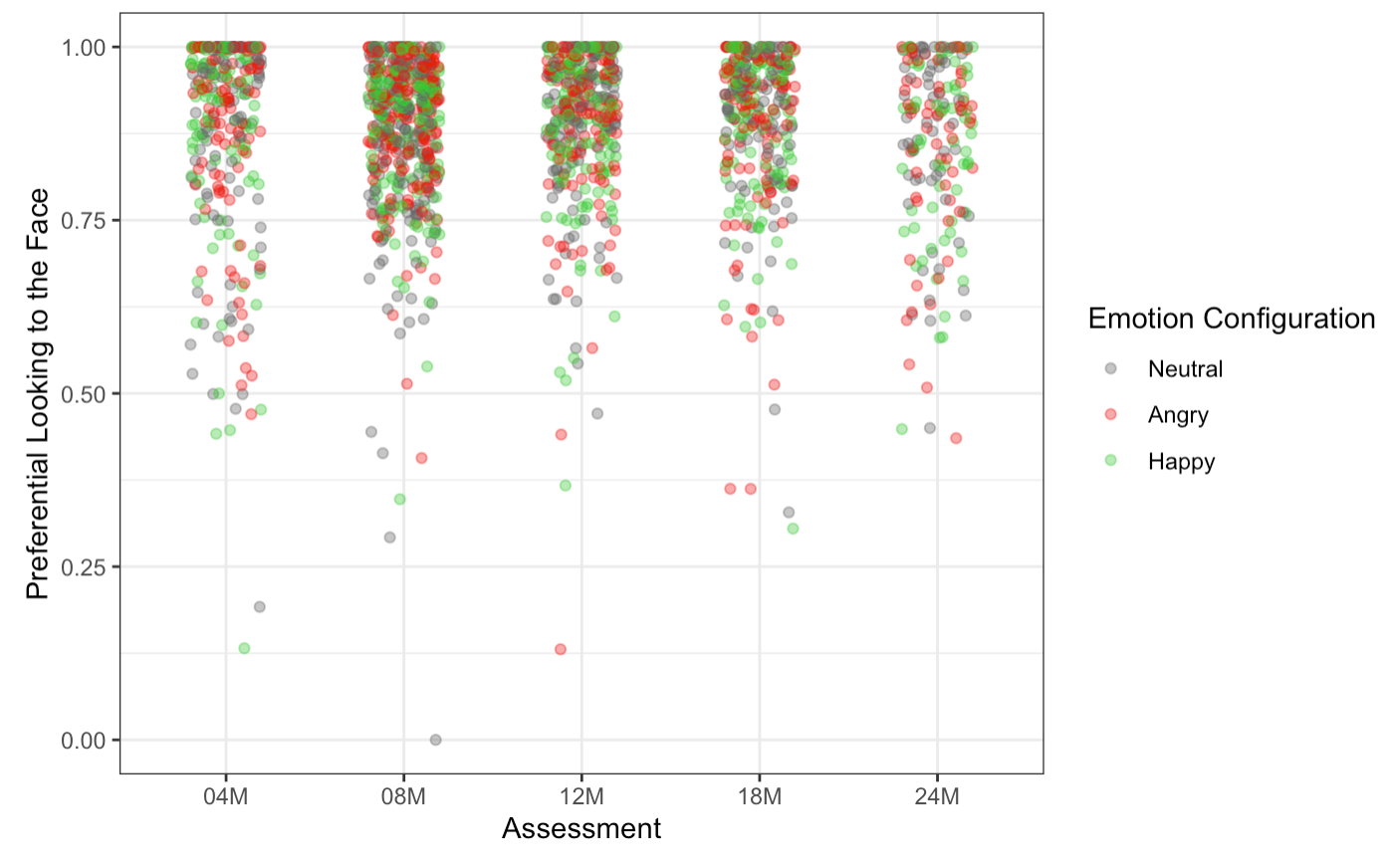


*Figure S6. Spread of Final Data from the Overlap Task, Preferential Looking to the Face (after cleaning for outliers and insufficient trials)*


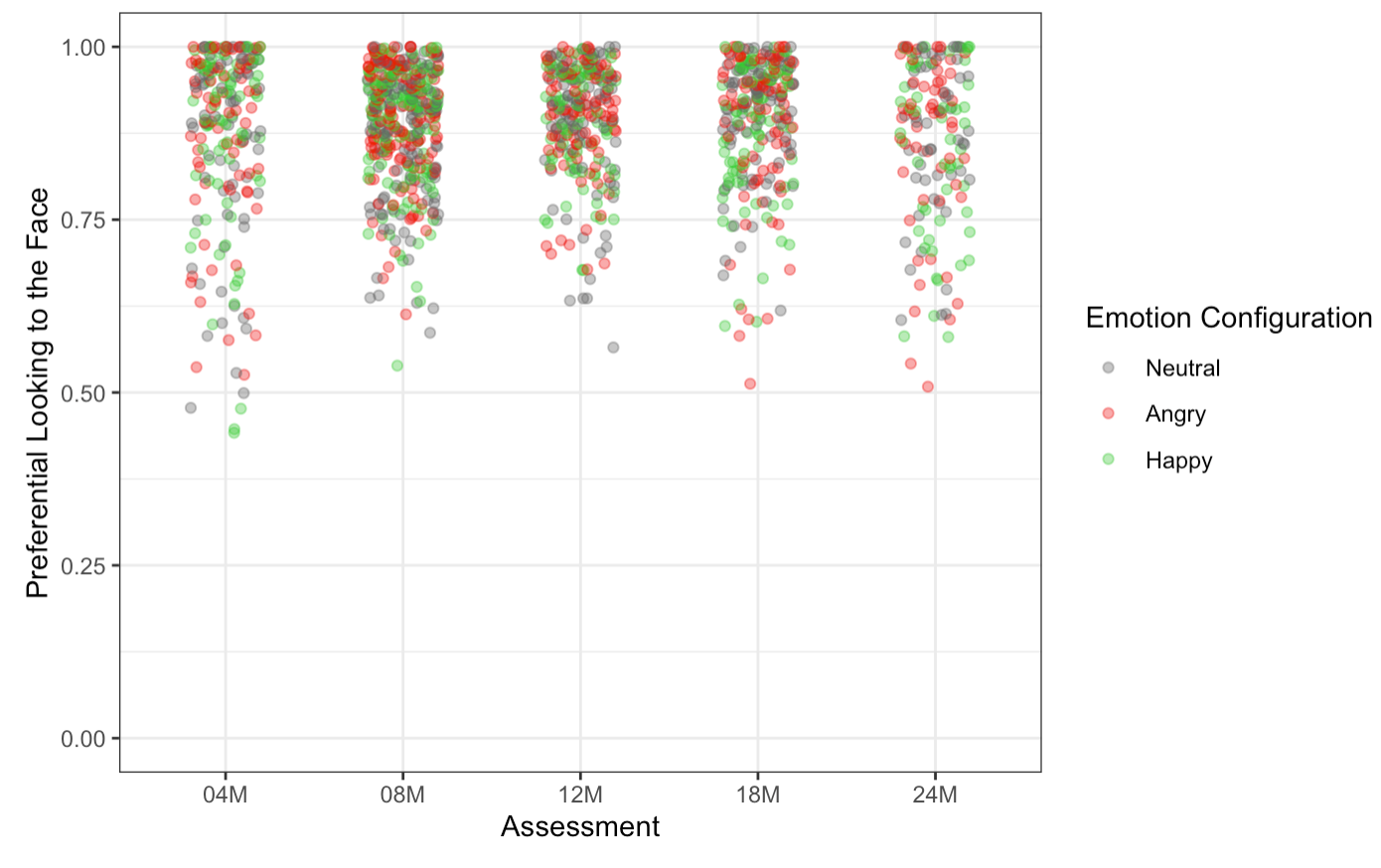

Supplement: Supplementary file 1 — Appendix S1 [file CDEV-93-e607-s001.docx]
