# Supplementary material for: Developmental patterns of affective attention across the first 2 years of life
Source: Child Dev. 2022 Jul 29;93(6):e607–21. doi: 10.1111/cdev.13831 (PMC9796239; doi:10.1111/cdev.13831)
Supplement: Supplementary file 2 — Appendix S2 [file CDEV-93-e607-s002.pdf]

Developmental Patterns of Affective Attention - Overlap Results

Lori B. Reider

01/28/2022

This file provides all code used for the analysis of the overlap data.

Relevant Packages

```
library(ggplot2)
library(lavaan)
library(lme4)
library(nlme)
library(psych)
library(lmerTest)
library(optimx)
library(tidyverse)

library(dplyr)
library(sjPlot)
library(corrplot)
```

```
latency <- read.csv("ovp_latency_11172021.csv", header=TRUE)
pref<- pref<- read.csv("ovp_prefscore12212021.csv", header = TRUE)
dwellprobe<- read.csv("ovp_dwellprobe_12202021.csv", header=TRUE)
```

```
pref$emotion_ov<- factor(pref$emotion_ov, levels = c("neutral", "angry", "happy"))
latency$emotion_ov<- factor(latency$emotion_ov, levels = c("neutral", "angry", "happy"))
dwellprobe$emotion_ov<- factor(dwellprobe$emotion_ov, levels = c("neutral", "angry", "happy"))



latency$time_recode<- as.numeric(latency$time_recode)
pref$time_recode<- as.numeric(pref$time_recode)
dwellprobe$time_recode<- as.numeric(dwellprobe$time_recode)


latency$n<- as.numeric(latency$n)
pref$n_facepref<- as.numeric(pref$n_facepref)
pref$n_probepref<- as.numeric(pref$n_probepref)
dwellprobe$cleaned_n<- as.numeric(dwellprobe$cleaned_n)


latency$assessment<- recode(latency$time_recode, '0' = "04M", '1' = "08M", '2' = "12M", '3'= "18M", '4'= "24M")
pref$assessment<- recode(pref$time_recode, '0' = "04M", '1' = "08M", '2' = "12M", '3'= "18M", '4'= "24M")
dwellprobe$assessment<- recode(dwellprobe$time_recode, '0' = "04M", '1' = "08M", '2' = "12M", '3'= "18M", '4'= "2
4M")

pref$emotion_ovfactor<- recode(pref$emotion_ov, '0' = "neutral", '1' = "angry", '2' = "happy")


# Function part of dependlab package
check_singularity <- function(lmerobj) {
  tt <- getME(lmerobj,"theta") #RE estimates
  ll <- getME(lmerobj,"lower") #lower bound on estimate

  #look for RE estimates that are very small (near zero) and the lower bound encompasses 0
  low_re <- tt[ll==0 & tt < 1e-2]
  return(low_re)
}
```

Descriptive Data

Raw data: Latency to Fixate Probe by Emotion (Angry, Happy, Neutral) and assessment (4,8,12,18,24 months)

```
latency%>%
  group_by(time_recode,emotion_ov)%>%
  summarize(mean(meanlatency, na.rm = TRUE),
            sd(meanlatency, na.rm = TRUE),
            min(meanlatency, na.rm = TRUE),
            max(meanlatency, na.rm = TRUE),
            n())

latency%>%
  group_by(time_recode,emotion_ov)%>%
  summarize(mean(n, na.rm = TRUE),
            sd(n, na.rm = TRUE),
            min(n, na.rm = TRUE),
            max(n, na.rm = TRUE),
            n())
```

Raw data: Preferential Looking to Probe by Emotion (Angry, Happy, Neutral) and assessment (4,8,12,18,24 months)

```
pref%>%
  group_by(time_recode,emotion_ov)%>%
  summarize(mean(mean_probeprefscore, na.rm = TRUE),
            sd(mean_probeprefscore, na.rm = TRUE),
            min(mean_probeprefscore, na.rm = TRUE),
            max(mean_probeprefscore, na.rm = TRUE),
            n())

pref%>%
  group_by(time_recode,emotion_ov)%>%
  summarize(mean(n, na.rm = TRUE),
            sd(n, na.rm = TRUE),
            min(n, na.rm = TRUE),
            max(n, na.rm = TRUE),
            n())
```

Raw data: Preferential Looking to Face by Emotion (Angry, Happy, Neutral) and assessment (4,8,12,18,24 months)

```
pref%>%
  group_by(time_recode,emotion_ov)%>%
  summarize(mean(mean_faceprefscore, na.rm = TRUE),
            sd(mean_faceprefscore, na.rm = TRUE),
            min(mean_faceprefscore, na.rm = TRUE),
            max(mean_faceprefscore, na.rm = TRUE),
            n())

pref%>%
  group_by(time_recode,emotion_ov)%>%
  summarize(mean(n, na.rm = TRUE),
            sd(n, na.rm = TRUE),
            min(n, na.rm = TRUE),
            max(n, na.rm = TRUE),
            n())
```

Raw data: Any Looks to Probe by Emotion (Angry, Happy, Neutral) and assessment (4,8,12,18,24 months)

```
dwellprobe%>%
  group_by(time_recode,emotion_ov)%>%
  summarize(mean(looktoprobe, na.rm = TRUE),
            sd(looktoprobe, na.rm = TRUE),
            min(looktoprobe, na.rm = TRUE),
            max(looktoprobe, na.rm = TRUE),
            n())
```

Descriptive Data: cleaned data (after removing outliers and insufficient number of trials)

Cleaned data: Latency to Fixate Probe by Emotion (Angry, Happy, Neutral) and assessment (4,8,12,18,24 months)

```r
latency%>%
  group_by(time_recode,emotion_ov)%>%
  summarize(mean(cleaned_meanlatency, na.rm = TRUE),
            sd(cleaned_meanlatency, na.rm = TRUE),
            min(cleaned_meanlatency, na.rm = TRUE),
            max(cleaned_meanlatency, na.rm = TRUE),
            n())

latency%>%
  group_by(time_recode,emotion_ov)%>%
  summarize(mean(cleaned_n, na.rm = TRUE),
            sd(cleaned_n, na.rm = TRUE),
            min(cleaned_n, na.rm = TRUE),
            max(cleaned_n, na.rm = TRUE),
            n())

#this resulted in the following data point loss:
  ## 4m datapoints: 51 angry, 47 happy, 60 neutral
  ## 8m datapoints: 116 angry, 118 happy, 122 neutral
  ## 12m datapoints: 92 angry, 92 happy, 92 neutral
  ## 18m datapoints: 73 angry, 69 happy, 72 neutral
  ## 24m datapoints: 42 angry, 46 happy, 42 neutral

#1134 datapoints were lost due to having an insufficient number of trials. All 30 outliers were also excluded bec
ause they also had an insufficient number of trials
```

Cleaned data: Preferential Looking to Face by Emotion (Angry, Happy, Neutral) and assessment (4,8,12,18,24 months)

```
pref%>%
  group_by(time_recode,emotion_ov)%>%
  summarize(mean(cleaned_mean_faceprefscore, na.rm = TRUE),
            sd(cleaned_mean_faceprefscore, na.rm = TRUE),
            min(cleaned_mean_faceprefscore, na.rm = TRUE),
            max(cleaned_mean_faceprefscore, na.rm = TRUE),
            n())

pref%>%
  group_by(time_recode,emotion_ov)%>%
  summarize(mean(n_facepref, na.rm = TRUE),
            sd(n_facepref, na.rm = TRUE),
            min(n_facepref, na.rm = TRUE),
            max(n_facepref, na.rm = TRUE),
            n())

#this resulted in the following data point loss:
  ## 4m datapoints: 18 angry, 17 happy, 14 neutral
  ## 8m datapoints:  43 angry, 37 happy, 36 neutral
  ## 12m datapoints: 28 angry, 29 happy, 33 neutral
  ## 18m datapoints: 13 angry, 12 happy, 12 neutral
  ## 24m datapoints: 7  angry, 7 happy, 9 neutral

#315 datapoints lost to insufficient number of trials, there were 25 outliers, and were already removed due to  i
nsufficient trials
```

Cleaned data: Preferential Looking to Probe by Emotion (Angry, Happy, Neutral) and assessment (4,8,12,18,24 months)

```
pref%>%
  group_by(time_recode,emotion_ov)%>%
  summarize(mean(cleaned_mean_probeprefscore, na.rm = TRUE),
            sd(cleaned_mean_probeprefscore, na.rm = TRUE),
            min(cleaned_mean_probeprefscore, na.rm = TRUE),
            max(cleaned_mean_probeprefscore, na.rm = TRUE),
            n())

pref%>%
  group_by(time_recode,emotion_ov)%>%
  summarize(mean(n_probepref, na.rm = TRUE),
            sd(n_probepref, na.rm = TRUE),
            min(n_probepref, na.rm = TRUE),
            max(n_probepref, na.rm = TRUE),
            n())


#this resulted in the following data point loss:
  ## 4m datapoints: 18 angry, 17 happy,  14 neutral
  ## 8m datapoints: 43 angry, 37 happy, 37 neutral
  ## 12m datapoints: 28 angry, 29 happy, 33 neutral
  ## 18m datapoints: 13 angry, 13 happy, 12 neutral
  ## 24m datapoints: 7 angry, 7 happy, 9 neutral

# 317 datapoints lost to insufficient number of trials, there were 26 outliers, and all were already removed due
 to insufficient trials
```

Data Visualizations

Visual Spread of latency to fixate probe data by emotion and assessment

Raw Data

```
#Plot longitudinal data (dot plots)- RAW LATENCIES
#spread of data at each timepoint

raw_latency_dot<- latency %>%
  ggplot(aes(x = assessment, y = meanlatency, color = emotion_ov, group = record_id)) +
  geom_point(position = position_jitter(w = .2), alpha = .4) +
  theme_bw() + # nice theme
  labs(x = "Assessment", y = "Latency to Fixate the Probe (ms)")+
  scale_color_manual(name= "Emotion Configuration",
                     labels = c("Neutral", "Angry", "Happy"),
                     values = c("grey40", "red2", "limegreen"))+ ylim(0,3200)

raw_latency_dot
```

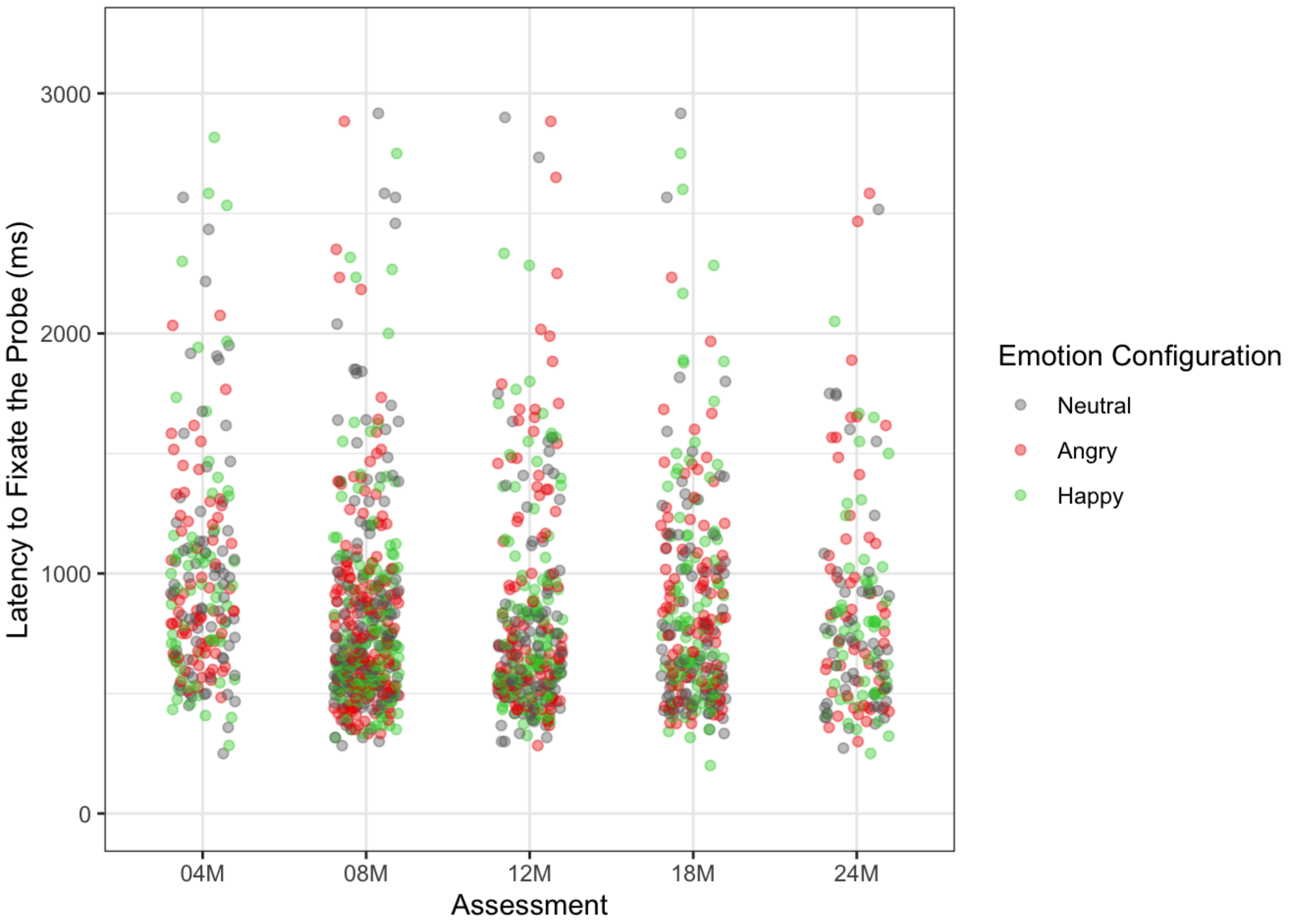

Cleaned Data

```
#Plot longitudinal data (dot plots)- CLEANED LATENCIES
#spread of data at each timepoint

clean_latency_dot<- latency %>%
  ggplot(aes(x = assessment, y = cleaned_meanlatency, color = emotion_ov, group = record_id)) +
  geom_point(position = position_jitter(w = .2), alpha = .4) +
  theme_bw() + # nice theme
  labs(x = "Assessment", y = "Latency to Fixate the Probe (ms)")+
  scale_color_manual(name= "Emotion Configuration",
                     labels = c("Neutral", "Angry", "Happy"),
                     values = c("grey40", "red2", "limegreen"))+ ylim(0,3200)

clean_latency_dot
```

```
## Warning: Removed 1134 rows containing missing values (geom_point).
```

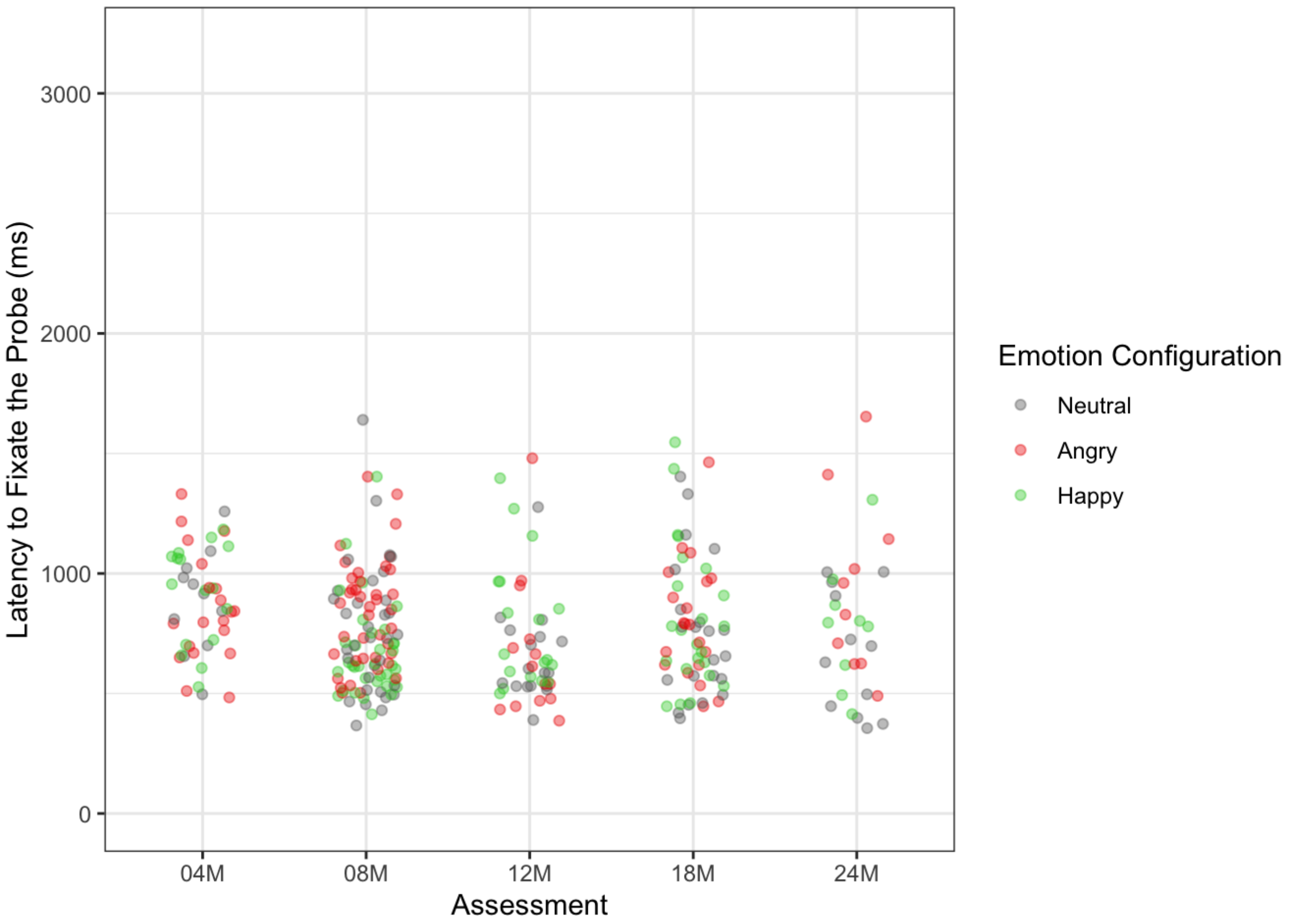

Visual Spread of preferential looking to the face data by emotion and assessment

Raw Data

```
#Plot longitudinal data (dot plots)- RAW PREF FACE
#spread of data at each timepoint

raw_facepref_dot<- pref %>%
  ggplot(aes(x = assessment, y = mean_faceprefscore, color = emotion_ov, group = record_id)) +
  geom_point(position = position_jitter(w = .2), alpha = .4) +
  theme_bw() + # nice theme
  labs(x = "Assessment", y = "Preferential Looking to the Face")+
  scale_color_manual(name= "Emotion Configuration",
                     labels = c("Neutral", "Angry", "Happy"),
                     values = c("grey40", "red2", "limegreen"))
raw_facepref_dot
```

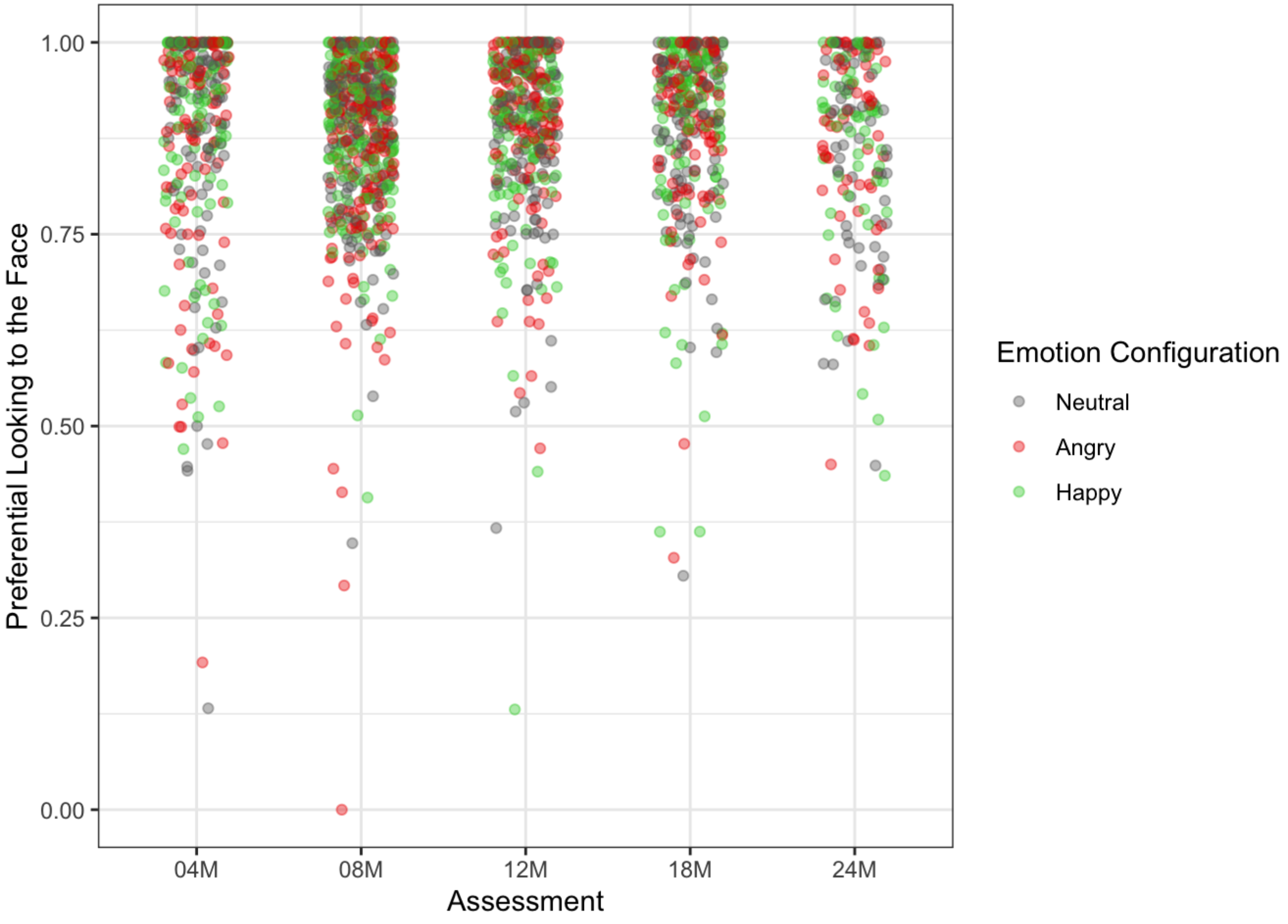

Cleaned Data

```
#Plot longitudinal data (dot plots)- CLEANED PREF FACE
#spread of data at each timepoint

clean_facepref_dot<- pref %>%
  ggplot(aes(x = assessment, y = cleaned_mean_faceprefscore, color = emotion_ov, group = record_id)) +
  geom_point(position = position_jitter(w = .2), alpha = .4) +
  theme_bw() + # nice theme
  labs(x = "Assessment", y = "Preferential Looking to the Face")+
  scale_color_manual(name= "Emotion Configuration",
                     labels = c("Neutral", "Angry", "Happy"),
                     values = c("grey40", "red2", "limegreen"))
clean_facepref_dot+ylim(0,1)
```

```
## Warning: Removed 367 rows containing missing values (geom_point).
```

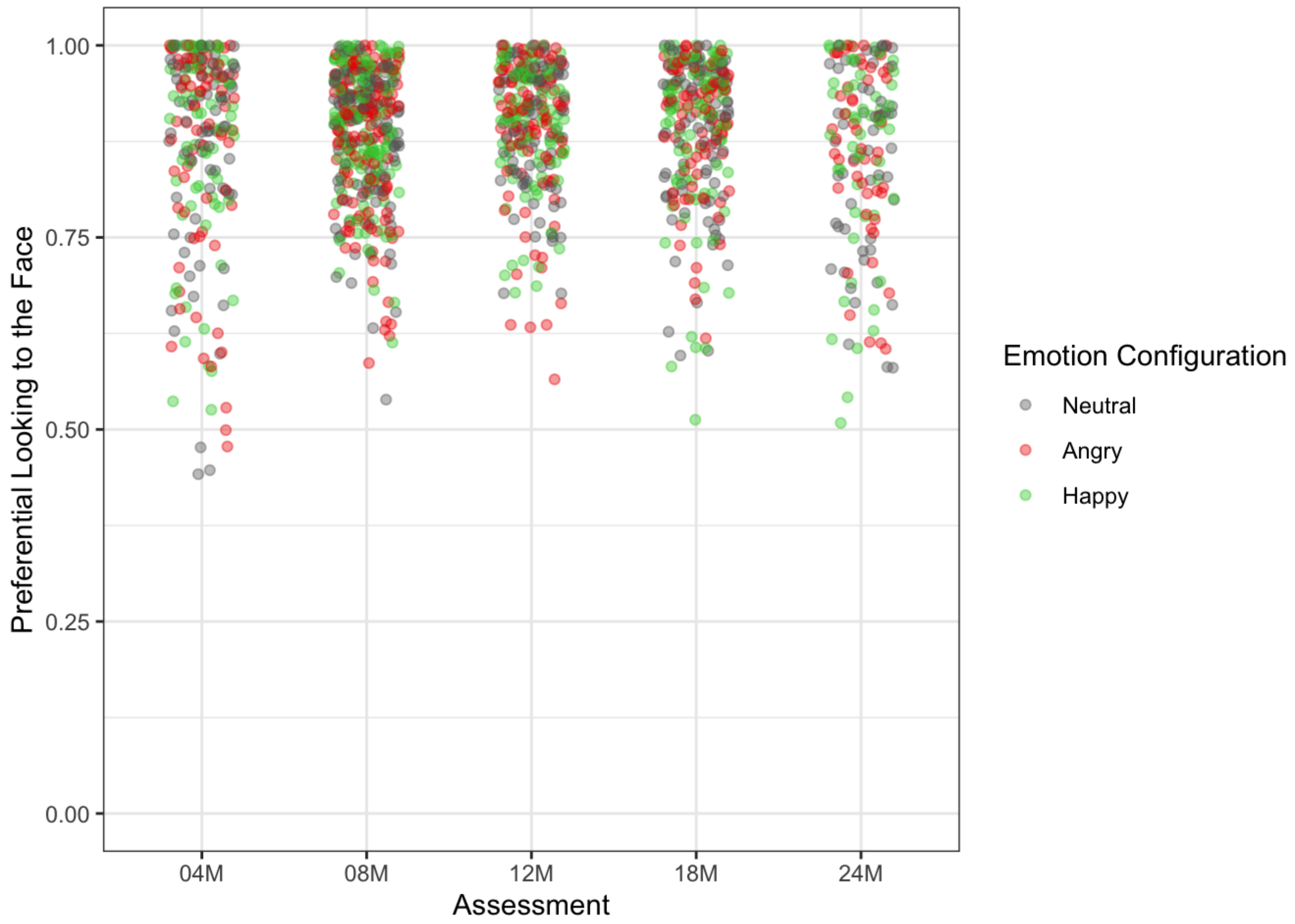

Visual Spread of preferential looking to the probe data by emotion and assessment

Raw Data

```
#Plot longitudinal data (dot plots)- RAW PREF PROBE
#spread of data at each timepoint

raw_probepref_dot<- pref %>%
  ggplot(aes(x = assessment, y = mean_probeprefscore, color = emotion_ov, group = record_id)) +
  geom_point(position = position_jitter(w = .2), alpha = .4) +
  theme_bw() + # nice theme
  labs(x = "Assessment", y = "Preferential Looking to the Probe")+
  scale_color_manual(name= "Emotion Configuration",
                     labels = c("Neutral", "Angry", "Happy"),
                     values = c("grey40", "red2", "limegreen"))
raw_probepref_dot
```

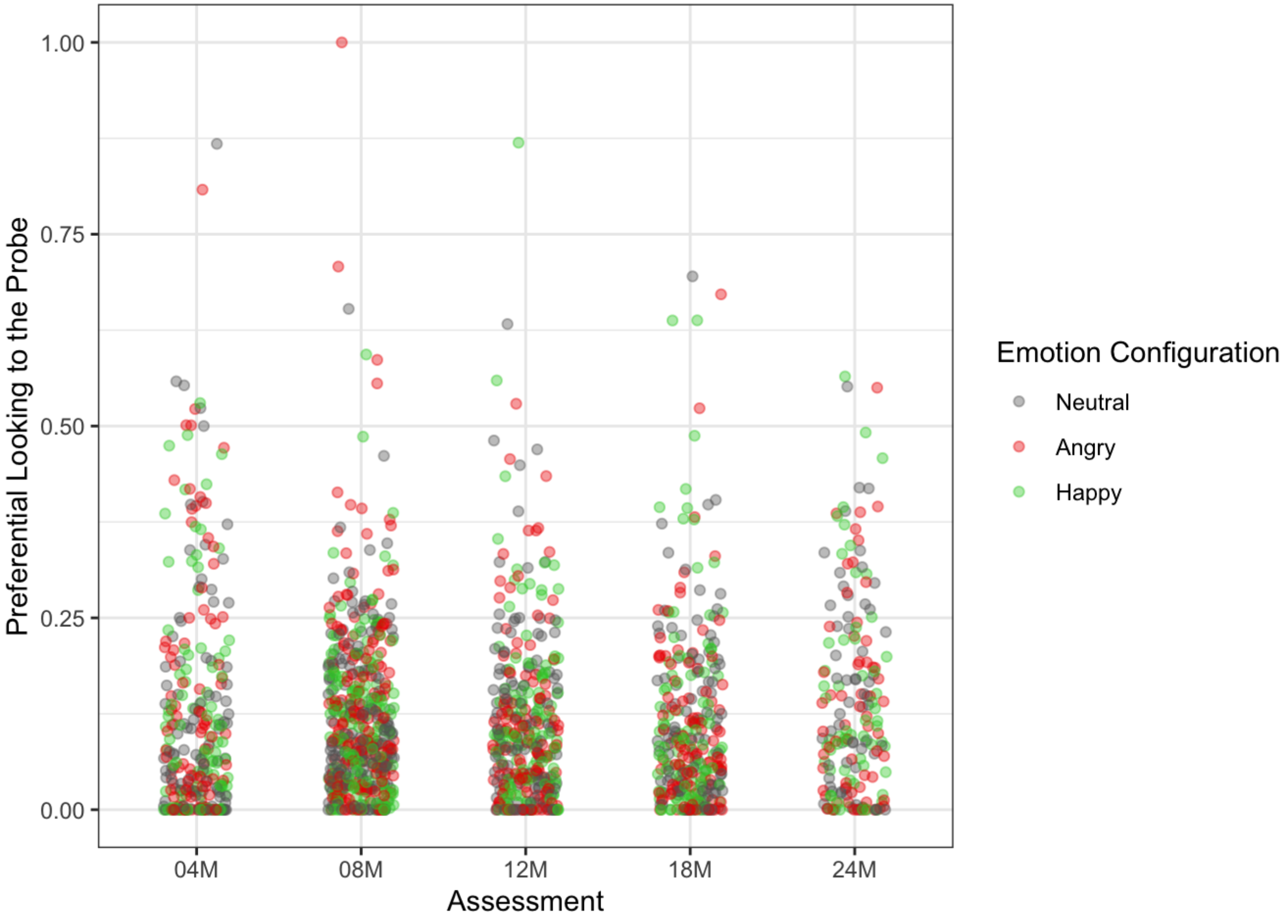

Cleaned Data

```
#Plot longitudinal data (dot plots)- CLEANED PREF PROBE
#spread of data at each timepoint

clean_probepref_dot<- pref %>%
  ggplot(aes(x = assessment, y = cleaned_mean_probeprefscore, color = emotion_ov, group = record_id)) +
  geom_point(position = position_jitter(w = .2), alpha = .4) +
  theme_bw() + # nice theme
  labs(x = "Assessment", y = "Preferential Looking to the Probe")+
  scale_color_manual(name= "Emotion Configuration",
                     labels = c("Neutral", "Angry", "Happy"),
                     values = c("grey40", "red2", "limegreen"))+ylim(0,1)
clean_probepref_dot
```

```
## Warning: Removed 365 rows containing missing values (geom_point).
```

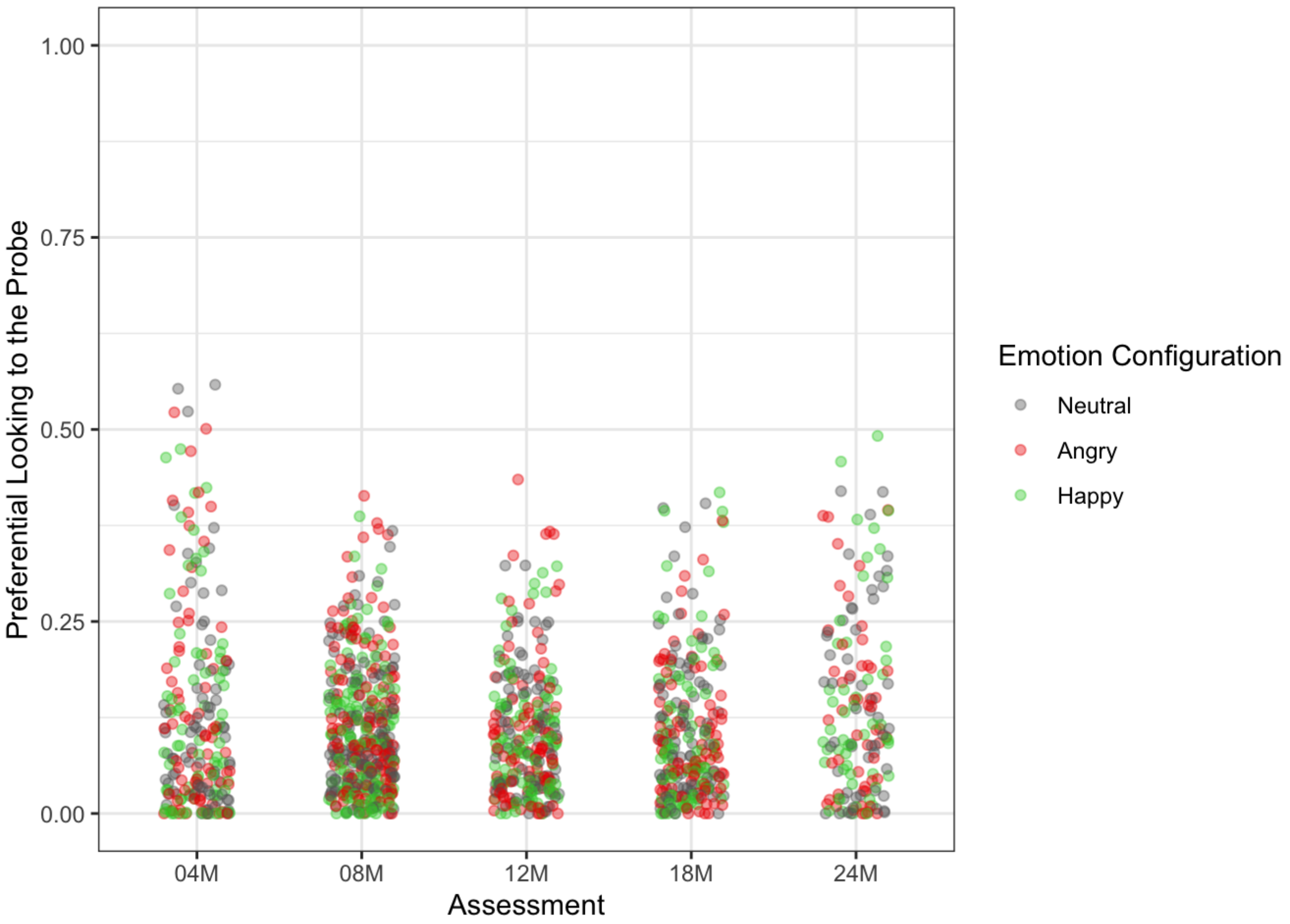

Visual Spread of any looks to the probe data by emotion and assessment

```
#Plot longitudinal data (dot plots)- RAW ANY LOOK TO PROBE
#spread of data at each timepoint

raw_lookprobe_dot<- dwellprobe %>%
  ggplot(aes(x = assessment, y = looktoprobe, color = emotion_ov, group = record_id)) +
  geom_point(position = position_jitter(w = .2), alpha = .4) +
  theme_bw() + # nice theme
  labs(x = "Assessment", y = "Number of trials infants looked to the probe")+
  scale_color_manual(name= "Emotion Configuration",
                     labels = c("Neutral", "Angry", "Happy"),
                     values = c("grey40", "red2", "limegreen"))
raw_lookprobe_dot
```

```
## Warning: Removed 165 rows containing missing values (geom_point).
```

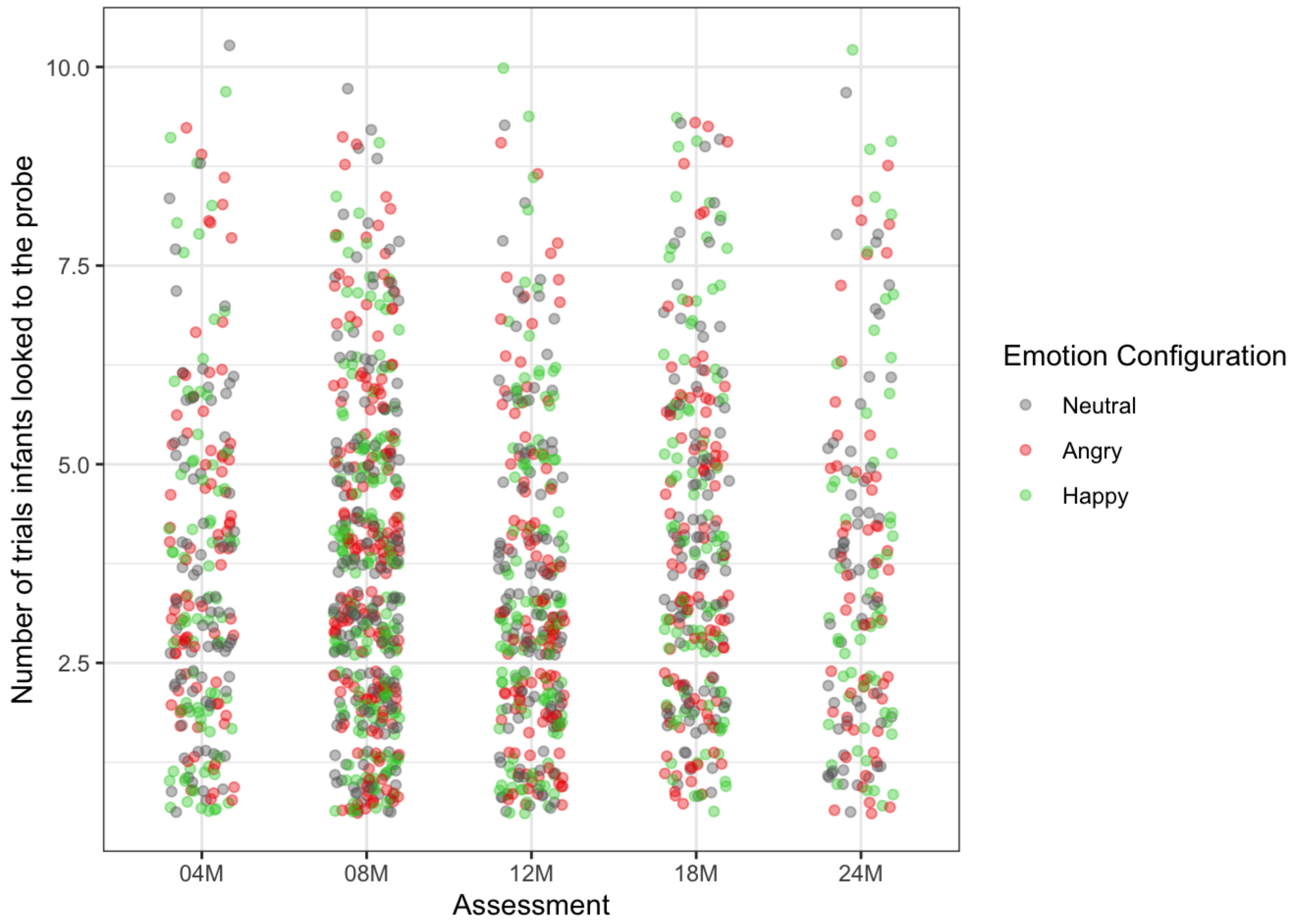

Correlations

Latency to Fixate the Probe

```
ovplat_reshape<-latency%>%
  select(record_id, time_recode, emotion_ov, cleaned_meanlatency, cleaned_n)%>%
  pivot_wider(names_from = c(time_recode, emotion_ov), values_from = c(cleaned_meanlatency, cleaned_n))

latencycorrel<-cor(ovplat_reshape, method = c("pearson"), use = "pairwise.complete.obs")
```

```
## Warning in cor(ovplat_reshape, method = c("pearson"), use =
## "pairwise.complete.obs"): the standard deviation is zero
```

```
corrplot(latencycorrel, method = 'shade', type= "lower")
```

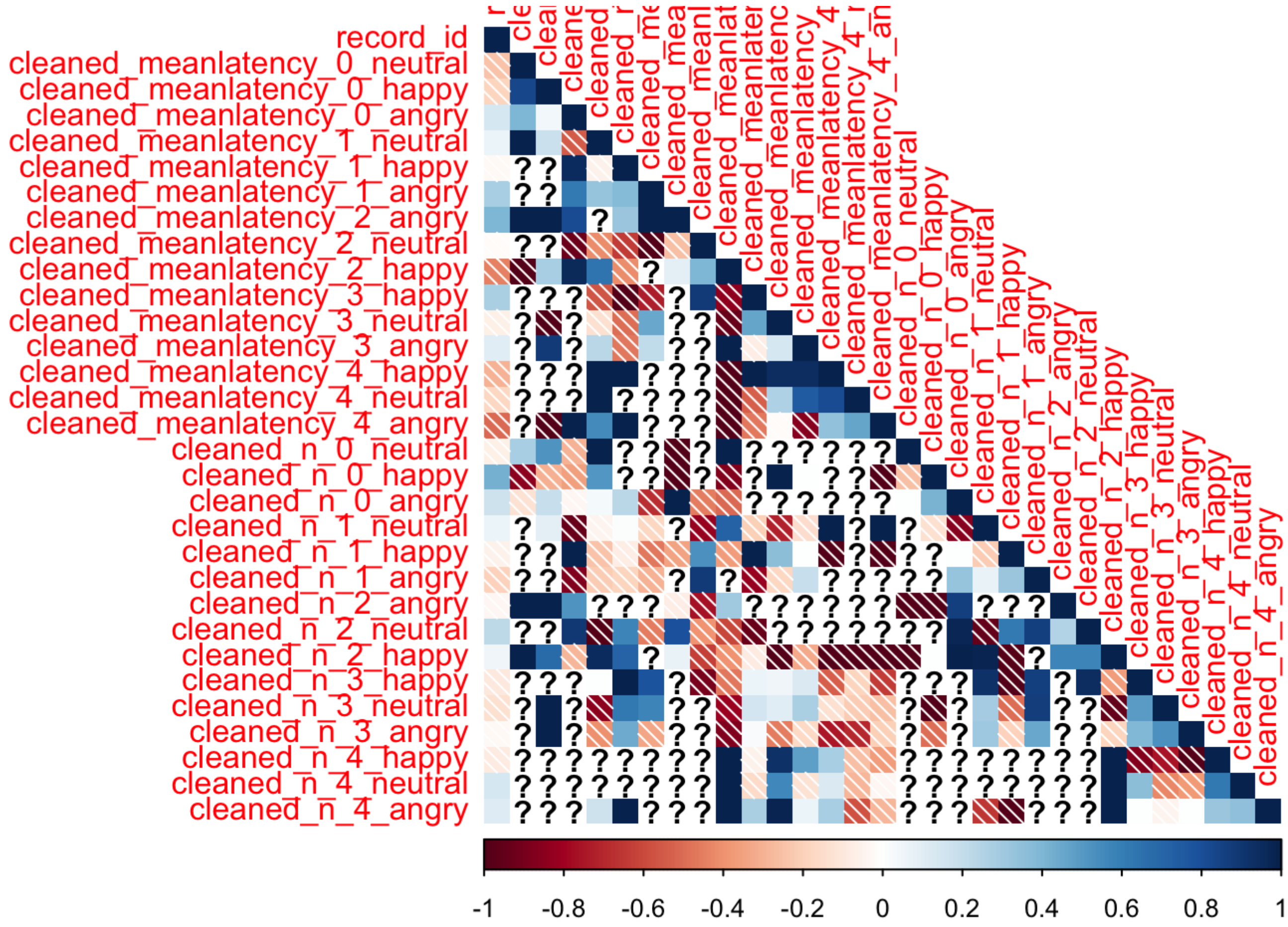

Preferential looking to the face

```
facepref_reshape<-pref%>%
  select(record_id, time_recode, emotion_ov, cleaned_mean_faceprefscore, n_facepref)%>%
  pivot_wider(names_from = c(time_recode, emotion_ov), values_from = c(cleaned_mean_faceprefscore,n_facepref))

ovpfaceprefcorrel<-cor(facepref_reshape, method = c("pearson"), use = "pairwise.complete.obs")
corrplot(ovpfaceprefcorrel, method = 'shade', type= "lower")
```

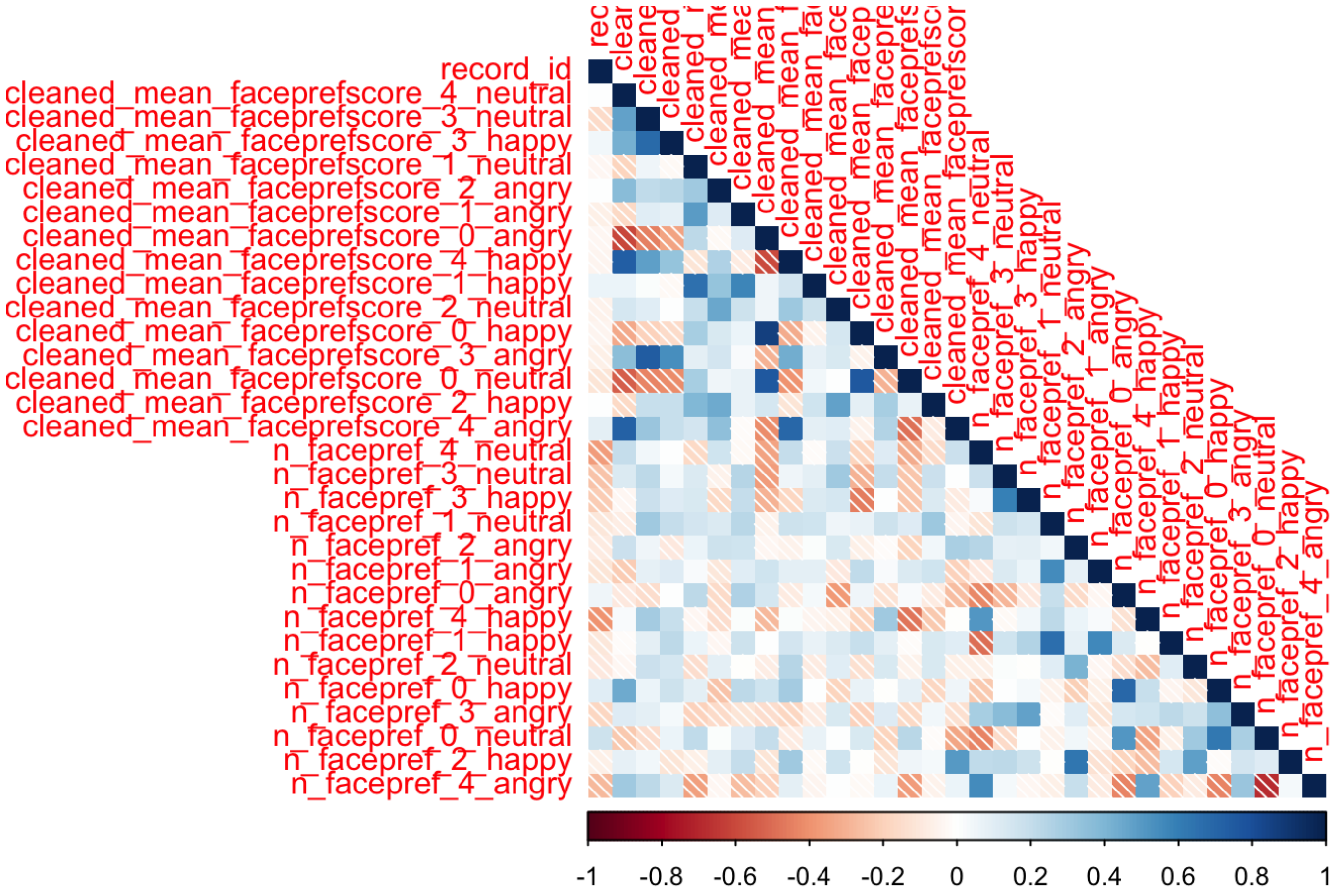

Preferential looking to the probe

```
probepref_reshape<-pref%>%
  select(record_id, time_recode, emotion_ov, cleaned_mean_probeprefscore, n_probepref)%>%
  pivot_wider(names_from = c(time_recode, emotion_ov), values_from = c(cleaned_mean_probeprefscore,n_probepref))

ovpprobeprefcorrel<-cor(probepref_reshape, method = c("pearson"), use = "pairwise.complete.obs")
corrplot(ovpprobeprefcorrel, method = 'shade', type= "lower")
```

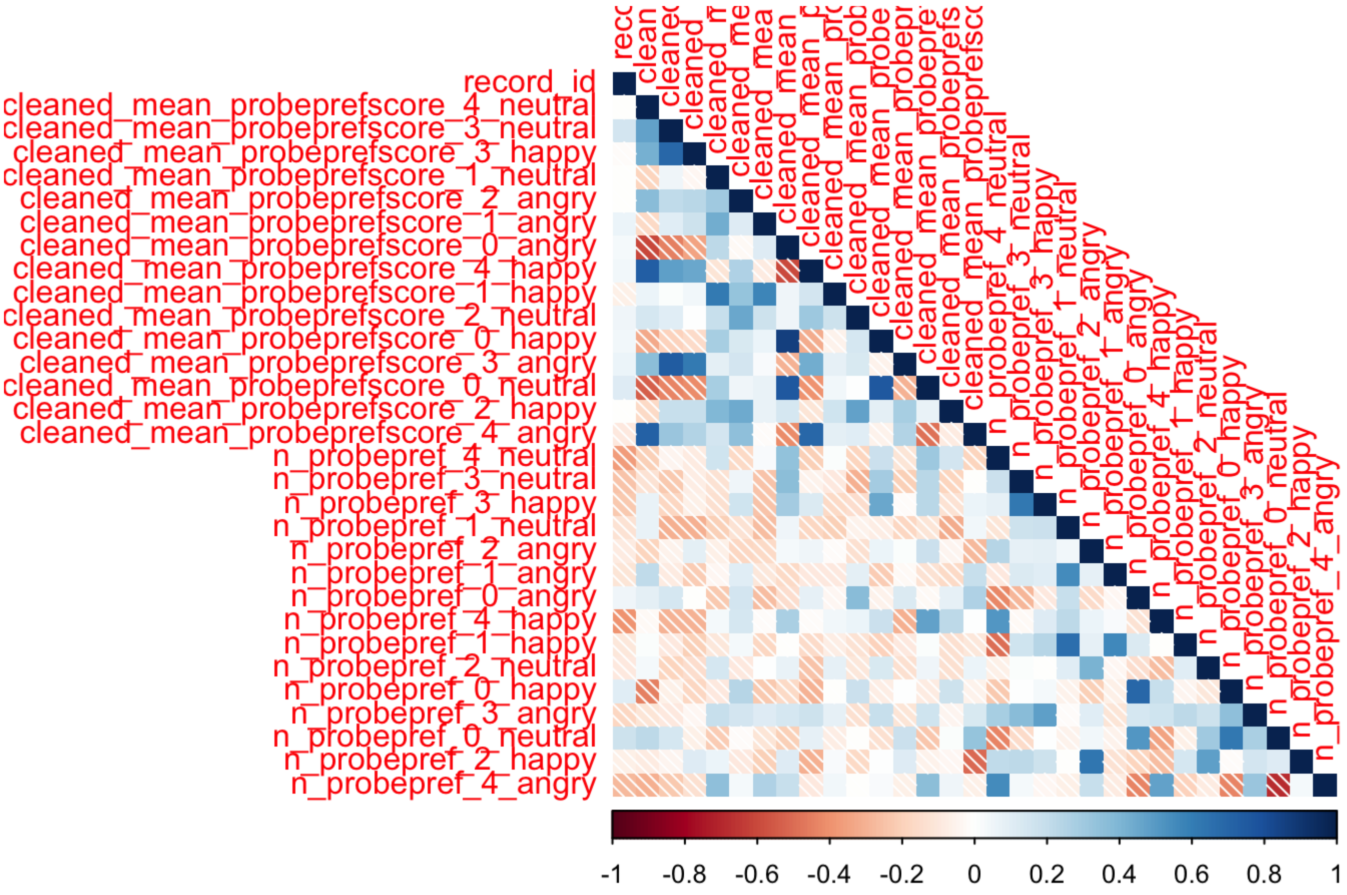

Multilevel Growth Curves: Overlap Task- Preferential looking to Face

Model lm0f: Intercept Only Model

```
lm0f <- lmer(cleaned_mean_faceprefscore~ 1 + (1|record_id), pref,
             control = lmerControl(optimizer ='optimx', optCtrl=list(method='L-BFGS-B')),
             REML = TRUE)
summary(lm0f)
```

```
## Linear mixed model fit by REML. t-tests use Satterthwaite's method [
## lmerModLmerTest]
## Formula: cleaned_mean_faceprefscore ~ 1 + (1 | record_id)
##    Data: pref
## Control: lmerControl(optimizer = "optimx", optCtrl = list(method = "L-BFGS-B"))
##
## REML criterion at convergence: -2683.1
##
## Scaled residuals:
##     Min      1Q  Median      3Q     Max
## -4.8393 -0.4572  0.1965  0.5815  2.6029
##
## Random effects:
##  Groups    Name        Variance Std.Dev.
##  record_id (Intercept) 0.003851 0.06206
##  Residual              0.006869 0.08288
## Number of obs: 1416, groups:  record_id, 265
##
## Fixed effects:
##              Estimate Std. Error        df t value Pr(>|t|)
## (Intercept) 8.883e-01  4.553e-03 2.399e+02   195.1   <2e-16 ***
## ---
## Signif. codes:  0 '***' 0.001 '**' 0.01 '*' 0.05 '.' 0.1 ' ' 1
```

```
confint(lm0f, method = "Wald")
```

```
##                 2.5 %    97.5 %
## .sig01             NA        NA
## .sigma             NA        NA
## (Intercept) 0.8793287 0.8971764
```

Model lm1f: Age as fixed and random effects in the model

```
lm1f <- lmer(cleaned_mean_faceprefscore ~ time_recode + (time_recode|record_id), pref,
             control = lmerControl(optimizer ='optimx', optCtrl=list(method='L-BFGS-B')),
             REML = TRUE)
summary(lm1f)
```

```
## Linear mixed model fit by REML. t-tests use Satterthwaite's method [
## lmerModLmerTest]
## Formula: cleaned_mean_faceprefscore ~ time_recode + (time_recode | record_id)
##    Data: pref
## Control: lmerControl(optimizer = "optimx", optCtrl = list(method = "L-BFGS-B"))
##
## REML criterion at convergence: -2921.8
##
## Scaled residuals:
##     Min      1Q  Median      3Q     Max
## -4.9125 -0.4657  0.1471  0.5417  2.8966
##
## Random effects:
##  Groups    Name        Variance Std.Dev. Corr
##  record_id (Intercept) 0.009393 0.09692
##            time_recode 0.002058 0.04536  -0.86
##  Residual              0.004884 0.06989
## Number of obs: 1416, groups:  record_id, 265
##
## Fixed effects:
##              Estimate Std. Error        df t value Pr(>|t|)
## (Intercept) 8.876e-01  7.634e-03 2.214e+02 116.273   <2e-16 ***
## time_recode 1.600e-03  3.818e-03 1.804e+02   0.419    0.676
## ---
## Signif. codes:  0 '***' 0.001 '**' 0.01 '*' 0.05 '.' 0.1 ' ' 1
##
## Correlation of Fixed Effects:
##             (Intr)
## time_recode -0.845
```

```
confint(lm1f, method = "Wald")
```

```
##                      2.5 %      97.5 %
## .sig01                  NA          NA
## .sig02                  NA          NA
## .sig03                  NA          NA
## .sigma                  NA          NA
## (Intercept)  0.872653616 0.902577847
## time_recode -0.005882198 0.009083044
```

```
plot_model(lm1f, type = "pred", show.data = F)
```

Model lm2f: Age and Emotion as fixed and random effects in the model:

```
lm2f <- lmer(cleaned_mean_faceprefscore ~ time_recode*emotion_ov + (time_recode+emotion_ov|record_id), pref,
             control = lmerControl(optimizer ='optimx', optCtrl=list(method='L-BFGS-B')),
              REML = TRUE)
```

```
## boundary (singular) fit: see ?isSingular
```

```
## Warning: Model failed to converge with 1 negative eigenvalue: -3.7e+03
```

```
 #summary(lm2f)
 #confint(lm2f, method = "Wald")

 if (isSingular(lm2f)) { print(check_singularity(lm2f)) }
```

```
##     record_id.time_recode record_id.emotion_ovhappy
##                         0                         0
```

```
#singularity issues
```

Runs but should ignore due to singularity. This model above throws a warning error. This model is likely over complicated given our dataset.

```
plot_model(lm2f, type = "int", mdrt.values = "meansd", show.data = T)
```

Model lm3f: Age as fixed and random, Emotion as fixed effects in model:

```
lm3f <- lmer(cleaned_mean_faceprefscore ~ time_recode*emotion_ov + (time_recode|record_id), pref,
             control = lmerControl(optimizer ='optimx', optCtrl=list(method='L-BFGS-B')),
             REML = TRUE)
summary(lm3f)
```

```
## Linear mixed model fit by REML. t-tests use Satterthwaite's method [
## lmerModLmerTest]
## Formula:
## cleaned_mean_faceprefscore ~ time_recode * emotion_ov + (time_recode |
##     record_id)
##    Data: pref
## Control: lmerControl(optimizer = "optimx", optCtrl = list(method = "L-BFGS-B"))
##
## REML criterion at convergence: -2891.3
##
## Scaled residuals:
##     Min      1Q  Median      3Q     Max
## -4.9315 -0.4826  0.1405  0.5435  2.9746
##
## Random effects:
##  Groups    Name        Variance Std.Dev. Corr
##  record_id (Intercept) 0.009374 0.09682
##            time_recode 0.002058 0.04536  -0.86
##  Residual              0.004874 0.06981
## Number of obs: 1416, groups:  record_id, 265
##
## Fixed effects:
##                               Estimate Std. Error         df t value Pr(>|t|)
## (Intercept)                  8.923e-01  8.857e-03  3.925e+02 100.746   <2e-16
## time_recode                 -1.688e-03  4.346e-03  3.013e+02  -0.388   0.6980
## emotion_ovangry             -1.515e-02  7.946e-03  1.020e+03  -1.907   0.0568
## emotion_ovhappy              7.009e-04  7.921e-03  1.024e+03   0.088   0.9295
## time_recode:emotion_ovangry  8.222e-03  3.624e-03  1.015e+03   2.269   0.0235
## time_recode:emotion_ovhappy  1.845e-03  3.629e-03  1.020e+03   0.508   0.6113
##
## (Intercept)                 ***
## time_recode
## emotion_ovangry             .
## emotion_ovhappy
## time_recode:emotion_ovangry *
## time_recode:emotion_ovhappy
## ---
## Signif. codes:  0 '***' 0.001 '**' 0.01 '*' 0.05 '.' 0.1 ' ' 1
##
```

```
## Correlation of Fixed Effects:
##                (Intr) tm_rcd emtn_vn emtn_vh tm_rcd:mtn_vn
## time_recode   -0.836
## emotn_vngry   -0.437  0.329
## emotn_vhppy   -0.442  0.336  0.492
## tm_rcd:mtn_vn  0.357 -0.411 -0.815  -0.403
## tm_rcd:mtn_vh  0.361 -0.417 -0.400  -0.815   0.497
```

```
confint(lm3f, method = "Wald")
```

```
##                                  2.5 %       97.5 %
## .sig01                              NA           NA
## .sig02                              NA           NA
## .sig03                              NA           NA
## .sigma                              NA           NA
## (Intercept)                0.874918045 0.9096356436
## time_recode               -0.010205354 0.0068293336
## emotion_ovangry           -0.030726108 0.0004225102
## emotion_ovhappy           -0.014823518 0.0162253266
## time_recode:emotion_ovangry  0.001119683 0.0153243141
## time_recode:emotion_ovhappy -0.005267459 0.0089570631
```

Plot of predicted model lm3

```
facefinal<- plot_model(lm3f, type = "int", mdrt.values = "meansd", show.data = F,colors=c("grey40", "red2", "lime
green"),axis.labels = "", title = "Final Model",
                         legend.title="Emotion Configuration")+
  labs(x = "Assessment", y = "Preferential Looking to the Face")

facefinal + theme_bw()+ylim(.80, 1.00)
```

```
## Scale for 'y' is already present. Adding another scale for 'y', which will
## replace the existing scale.
```

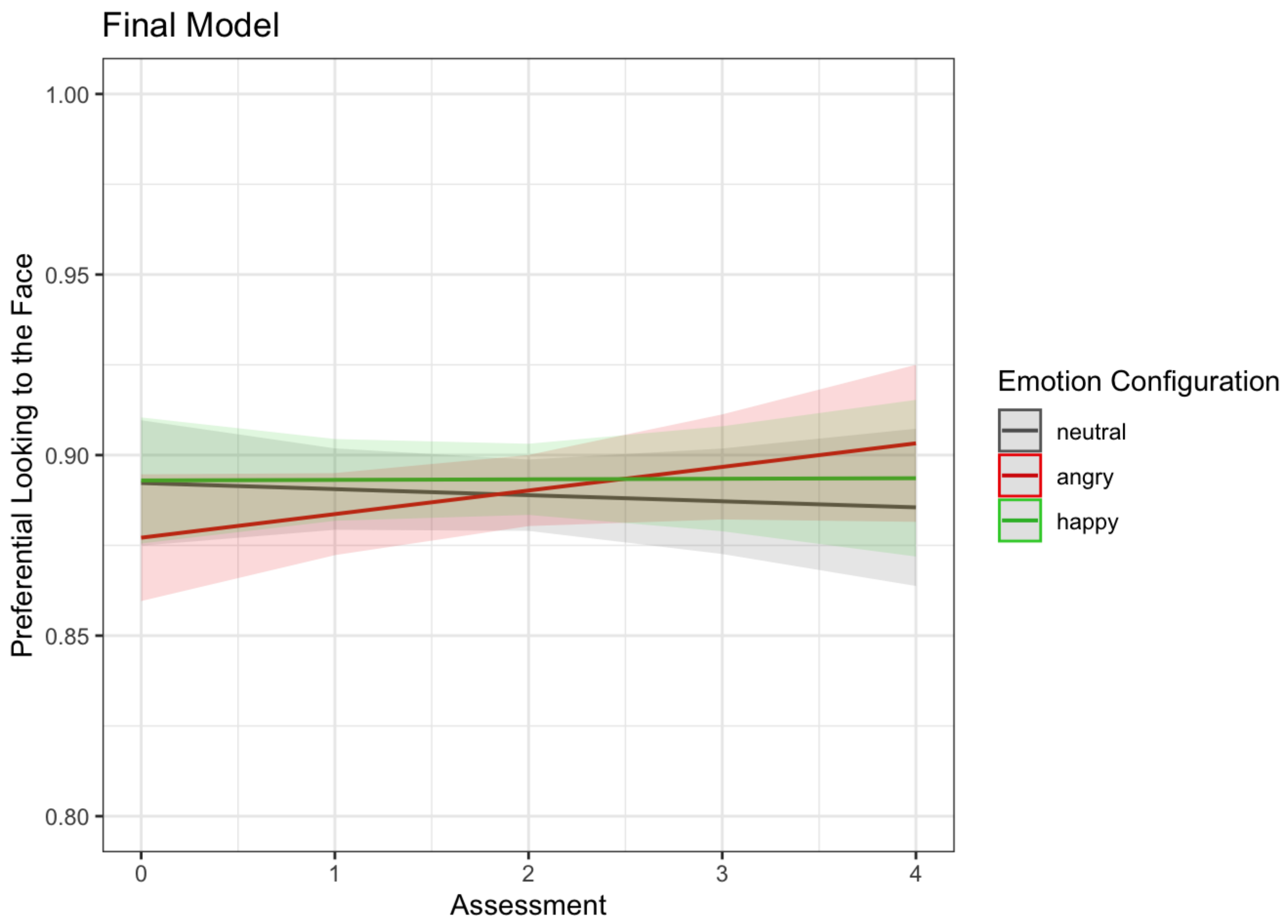

ANOVA Comparison Test of Model Fit:

```
anova(lm0f, lm1f, lm3f)
```

```
## refitting model(s) with ML (instead of REML)
```

```
## Data: pref
## Models:
## lm0f: cleaned_mean_faceprefscore ~ 1 + (1 | record_id)
## lm1f: cleaned_mean_faceprefscore ~ time_recode + (time_recode | record_id)
## lm3f: cleaned_mean_faceprefscore ~ time_recode * emotion_ov + (time_recode | record_id)
##      npar     AIC     BIC logLik deviance    Chisq Df Pr(>Chisq)
## lm0f    3 -2686.0 -2670.3 1346.0  -2692.0
## lm1f    6 -2928.3 -2896.8 1470.1  -2940.3 248.2510  3     <2e-16 ***
## lm3f   10 -2927.1 -2874.5 1473.5  -2947.1   6.7587  4     0.1492
## ---
## Signif. codes:  0 '***' 0.001 '**' 0.01 '*' 0.05 '.' 0.1 ' ' 1
```

Based on the study design, we selected Model 3 as the final model.

Follow-up paired samples t-test to examine when a AB to threat is present

```
t.test(facepref_reshape$cleaned_mean_faceprefscore_0_neutral, facepref_reshape$cleaned_mean_faceprefscore_0_angr
y, paired = TRUE, alternative = "two.sided") #4-month
```

```
##
##  Paired t-test
##
## data:  facepref_reshape$cleaned_mean_faceprefscore_0_neutral and facepref_reshape$cleaned_mean_faceprefscore_0
_angry
## t = 0.74157, df = 76, p-value = 0.4606
## alternative hypothesis: true difference in means is not equal to 0
## 95 percent confidence interval:
##  -0.01347471  0.02946132
## sample estimates:
## mean of the differences
##             0.007993308
```

```
t.test(facepref_reshape$cleaned_mean_faceprefscore_1_neutral, facepref_reshape$cleaned_mean_faceprefscore_1_angr
y, paired = TRUE, alternative = "two.sided") #8-month
```

```
##
##  Paired t-test
##
## data:  facepref_reshape$cleaned_mean_faceprefscore_1_neutral and facepref_reshape$cleaned_mean_faceprefscore_1
_angry
## t = 0.99626, df = 126, p-value = 0.321
## alternative hypothesis: true difference in means is not equal to 0
## 95 percent confidence interval:
##  -0.007763174  0.023503628
## sample estimates:
## mean of the differences
##             0.007870227
```

```
t.test(facepref_reshape$cleaned_mean_faceprefscore_2_neutral, facepref_reshape$cleaned_mean_faceprefscore_2_angr
y, paired = TRUE, alternative = "two.sided") #12-month
```

```
##
##  Paired t-test
##
## data:  facepref_reshape$cleaned_mean_faceprefscore_2_neutral and facepref_reshape$cleaned_mean_faceprefscore_2
_angry
## t = -0.61249, df = 85, p-value = 0.5419
## alternative hypothesis: true difference in means is not equal to 0
## 95 percent confidence interval:
##  -0.02312795  0.01223452
## sample estimates:
## mean of the differences
##            -0.005446715
```

```
t.test(facepref_reshape$cleaned_mean_faceprefscore_3_neutral, facepref_reshape$cleaned_mean_faceprefscore_3_angr
y, paired = TRUE, alternative = "two.sided") #18-month
```

```
##
##  Paired t-test
##
## data:  facepref_reshape$cleaned_mean_faceprefscore_3_neutral and facepref_reshape$cleaned_mean_faceprefscore_3
_angry
## t = -1.2623, df = 89, p-value = 0.2101
## alternative hypothesis: true difference in means is not equal to 0
## 95 percent confidence interval:
##  -0.021593374  0.004815961
## sample estimates:
## mean of the differences
##            -0.008388707
```

```
t.test(facepref_reshape$cleaned_mean_faceprefscore_4_neutral, facepref_reshape$cleaned_mean_faceprefscore_4_angr
y, paired = TRUE, alternative = "two.sided") #24-month
```

```
##
##  Paired t-test
##
## data:  facepref_reshape$cleaned_mean_faceprefscore_4_neutral and facepref_reshape$cleaned_mean_faceprefscore_4
_angry
## t = -2.216, df = 50, p-value = 0.03127
## alternative hypothesis: true difference in means is not equal to 0
## 95 percent confidence interval:
##  -0.051882983 -0.002547543
## sample estimates:
## mean of the differences
##             -0.02721526
```

```
#means and SDs for sig t-test
mean(facepref_reshape$cleaned_mean_faceprefscore_4_neutral, na.rm=TRUE)
```

```
## [1] 0.854381
```

```
sd(facepref_reshape$cleaned_mean_faceprefscore_4_neutral, na.rm=TRUE)
```

```
## [1] 0.122579
```

```
mean(facepref_reshape$cleaned_mean_faceprefscore_4_angry, na.rm=TRUE)
```

```
## [1] 0.8742898
```

```
sd(facepref_reshape$cleaned_mean_faceprefscore_4_angry, na.rm=TRUE)
```

```
## [1] 0.1126539
```
