# Supplementary material for: Developmental patterns of affective attention across the first 2 years of life
Source: Child Dev. 2022 Jul 29;93(6):e607–21. doi: 10.1111/cdev.13831 (PMC9796239; doi:10.1111/cdev.13831)
Supplement: Supplementary file 3 — Appendix S3 [file CDEV-93-e607-s003.pdf]

Developmental Patterns of Affective Attention - Vigilance Results

Lori B. Reider

01/28/2022

This file provides all code used for the analysis of the vigilance data.

Relevant Packages

```
library(ggplot2)
library(lavaan)
library(lme4)
library(nlme)
library(psych)
library(lmerTest)
library(optimx)
library(tidyverse)

library(dplyr)
library(sjPlot)
library(corrplot)
```

```
vig <- read.csv("vigilance_09142021.csv", header=TRUE)
reshaped_vig<- read.csv("vig_reshape_09162021_latency.csv", header= TRUE)
```

```
#format variables
vig$timepoint_recode<- as.numeric(vig$timepoint_recode)
vig$emotion_coded<- as.factor(vig$emotion_coded)
vig$emotion_factor<- recode(vig$emotion_coded, '0' = "neutral", '1' = "angry", '2' = "happy")
timepoint_asfactor<- recode(vig$timepoint_recode, '0' = "04M", '1' = "08M", '2' = "12M", '3'= "18M", '4'= "24M")



# Function part of dependlab package
check_singularity <- function(lmerobj) {
  tt <- getME(lmerobj,"theta") #RE estimates
  ll <- getME(lmerobj,"lower") #lower bound on estimate

  #look for RE estimates that are very small (near zero) and the lower bound encompasses 0
  low_re <- tt[ll==0 & tt < 1e-2]
  return(low_re)
}
```

Descriptive Data

Raw data: Latency to Fixate Emotion (Angry, Happy, Neutral) by assessment (4,8,12,18,24 months)

Note- coding of Time/Assessment: 0=4M, 1=8M, 2=12M, 3=18M, 4=24M

```
describeBy(vig$latency,list(vig$timepoint_recode,vig$emotion_factor))
describeBy(vig$num_trials,list(vig$timepoint_recode,vig$emotion_factor))
```

Cleaned Data: Latency to Fixate Emotion (Angry, Happy, Neutral) by assessment (4,8,12,18,24 months)

Datapoints defined by an insufficient number of trials or as outliers were removed in the clean dataset

```
describeBy(vig$latency_cleaned,list(vig$timepoint_recode,vig$emotion_factor))
describeBy(vig$num_trials_cleaned,list(vig$timepoint_recode,vig$emotion_factor))


# We lost 404 datapoints due to having a low number of trials.
#Of these, 25 were outliers. An additional 4 outliers were removed that had enough trials.
#Altogether, we lost 408 datapoints.

# Data loss by emotion and assessment:
# 4M:  35 neutral, 35 angry, 33 happy
# 8M:  45 neutral, 45 angry, 40 happy
# 12M: 32 neutral, 28 angry, 32 happy
# 18M: 18 neutral, 16 angry, 17 happy
# 24M:  9 neutral, 13 angry, 9 happy
```

Correlations on all outcome latency variables

```
vigcorrel<-cor(reshaped_vig, method = c("pearson"), use = "pairwise.complete.obs")
corrplot(vigcorrel, method = 'shade', type= "lower")
```

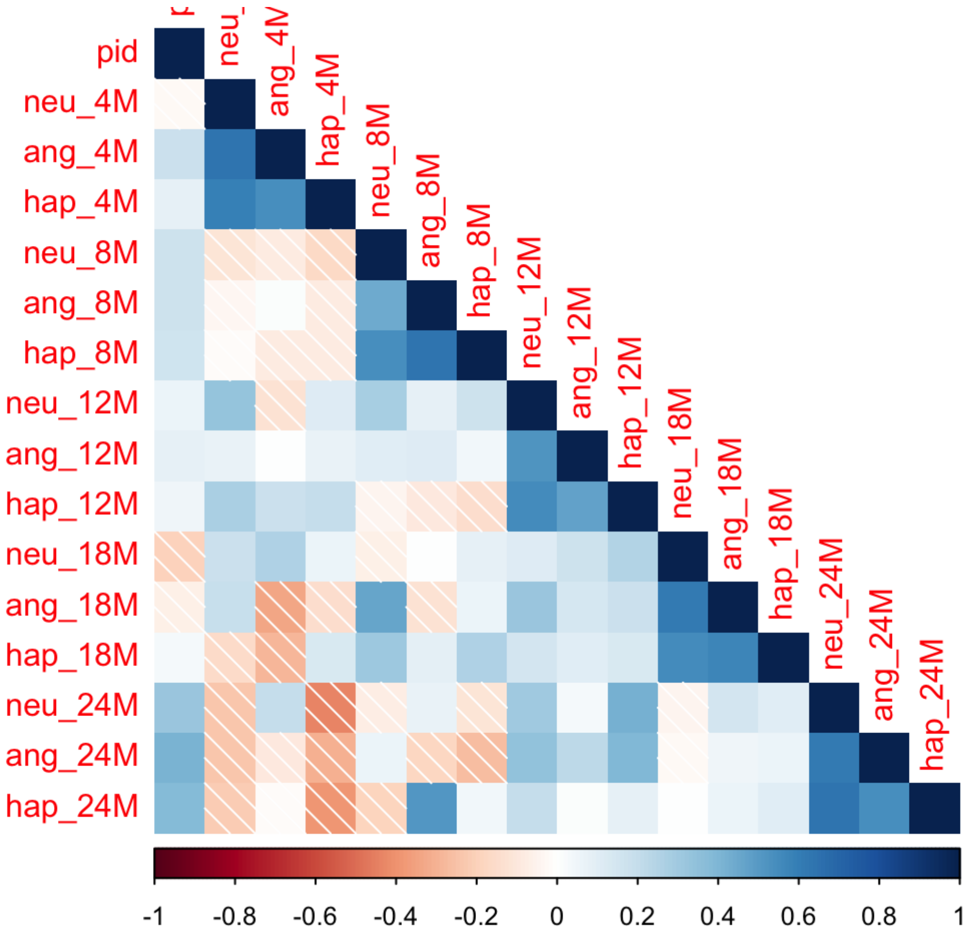

Data Visualizations

Visual Spread of the Raw latency data by emotion and assessment

```
#Plot longitudinal data (dot plots)
#spread of data at each timepoint

vig %>%
  ggplot(aes(x = timepoint_asfactor, y = latency, color = emotion_coded, group = pid)) +
  geom_point(position = position_jitter(w = .2), alpha = .4) +
    theme_bw() + # nice theme
  labs(x = "Assessment", y = "Latency to Fixate the Face (ms)")+
  scale_color_manual(name= "Emotion Configuration",
                     labels = c("Neutral", "Angry", "Happy"),
                     values = c("grey40", "red2", "limegreen"))+
  scale_x_discrete(labels =c("0" = "04M", "1" = "08M", "2" = "12M", "3" = "18M", "4"= "24M"))+ ylim(0,3500)
```

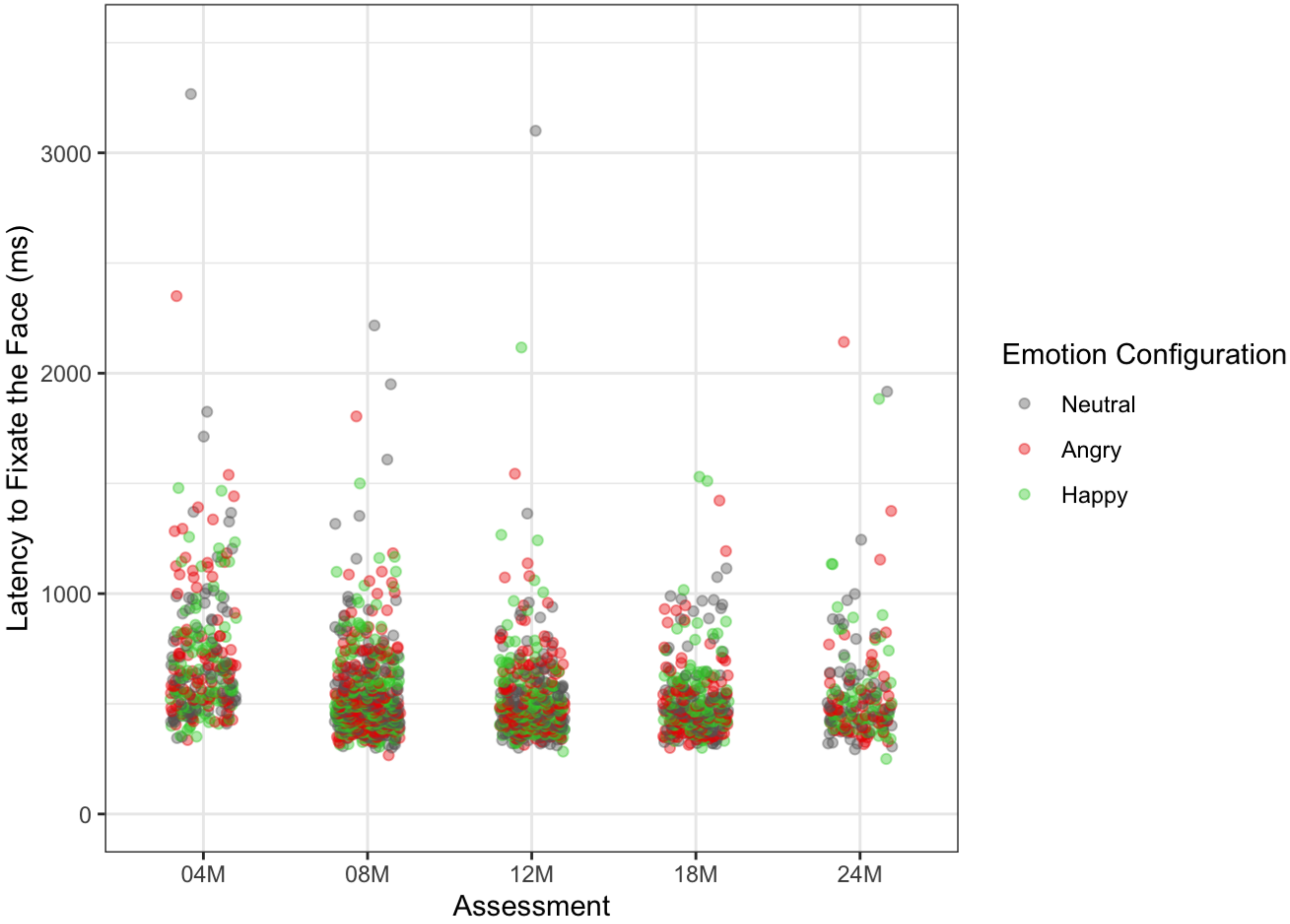

Visual Spread of the cleaned latency data by emotion and assessment

```
vig %>%
  ggplot(aes(x = timepoint_asfactor, y = latency_cleaned, color = emotion_coded, group = pid)) +
  geom_point(position = position_jitter(w = .2), alpha = .4) +
    theme_bw() + # nice theme
  labs(x = "Assessment", y = "Latency to Fixate the Face (ms)")+
  scale_color_manual(name= "Emotion Configuration",
                     labels = c("Neutral", "Angry", "Happy"),
                     values = c("grey40", "red2", "limegreen"))+
  scale_x_discrete(labels =c("0" = "04M", "1" = "08M", "2" = "12M", "3" = "18M", "4"= "24M"))+ ylim(0,3500)
```

```
## Warning: Removed 408 rows containing missing values (geom_point).
```

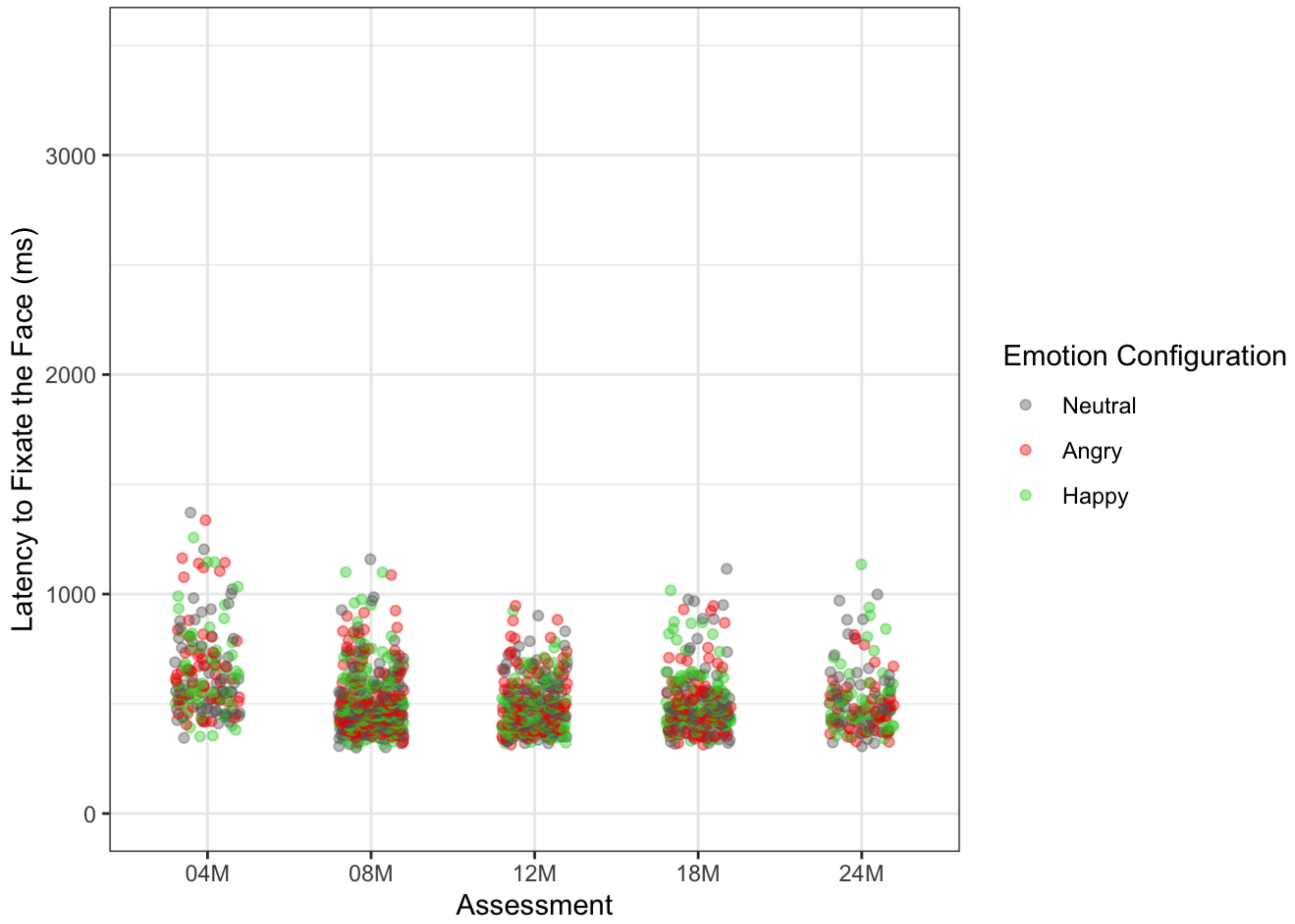

Multilvel Growth Curves: Vigilance Task

#Latency to Fixate Emotion Face Configurations (cleaned data)

Model lm0: Intercept Only Model

```
lm0b <- lmer(latency_cleaned~ 1 + (1|pid), vig,
             control = lmerControl(optimizer ='optimx', optCtrl=list(method='L-BFGS-B')),
             REML = TRUE)
summary(lm0b)
```

```
## Linear mixed model fit by REML. t-tests use Satterthwaite's method [
## lmerModLmerTest]
## Formula: latency_cleaned ~ 1 + (1 | pid)
##    Data: vig
## Control: lmerControl(optimizer = "optimx", optCtrl = list(method = "L-BFGS-B"))
##
## REML criterion at convergence: 17787.3
##
## Scaled residuals:
##     Min      1Q  Median      3Q     Max
## -2.2212 -0.5816 -0.1965  0.3586  5.3469
##
## Random effects:
##  Groups   Name        Variance Std.Dev.
##  pid      (Intercept)  8503     92.21
##  Residual             18365    135.52
## Number of obs: 1382, groups:  pid, 260
##
## Fixed effects:
##             Estimate Std. Error      df t value Pr(>|t|)
## (Intercept)  529.854      7.008 215.186    75.6   <2e-16 ***
## ---
## Signif. codes:  0 '***' 0.001 '**' 0.01 '*' 0.05 '.' 0.1 ' ' 1
```

```
confint(lm0b, method = "Wald")
```

```
##               2.5 % 97.5 %
## .sig01           NA     NA
## .sigma           NA     NA
## (Intercept) 516.118 543.59
```

Model lm1: Age as fixed and random effects in the model

```r
lm1b <- lmer(latency_cleaned ~ timepoint_recode + (timepoint_recode|pid), vig,
             control = lmerControl(optimizer ='optimx', optCtrl=list(method='L-BFGS-B')),
             REML = TRUE)
summary(lm1b)
```

```
## Linear mixed model fit by REML. t-tests use Satterthwaite's method [
## lmerModLmerTest]
## Formula: latency_cleaned ~ timepoint_recode + (timepoint_recode | pid)
##    Data: vig
## Control: lmerControl(optimizer = "optimx", optCtrl = list(method = "L-BFGS-B"))
##
## REML criterion at convergence: 17630.8
##
## Scaled residuals:
##     Min      1Q  Median      3Q     Max
## -2.9159 -0.5838 -0.1730  0.3869  4.1055
##
## Random effects:
##  Groups   Name             Variance Std.Dev. Corr
##  pid      (Intercept)      23478    153.23
##           timepoint_recode  4345     65.92   -0.90
##  Residual                  14818    121.73
## Number of obs: 1382, groups:  pid, 260
##
## Fixed effects:
##                  Estimate Std. Error      df t value Pr(>|t|)
## (Intercept)       557.475     12.691 206.552  43.927  < 2e-16 ***
## timepoint_recode  -23.573      5.915 154.605  -3.985 0.000104 ***
## ---
## Signif. codes:  0 '***' 0.001 '**' 0.01 '*' 0.05 '.' 0.1 ' ' 1
##
## Correlation of Fixed Effects:
##             (Intr)
## timepnt_rcd -0.867
```

```r
confint(lm1b, method = "Wald")
```

```
##                       2.5 %    97.5 %
## .sig01                   NA        NA
## .sig02                   NA        NA
## .sig03                   NA        NA
## .sigma                   NA        NA
## (Intercept)      532.60147 582.34855
## timepoint_recode -35.16651 -11.97957
```

Plot of predicted model lm1

```
plot_model(lm1b, type = "pred", show.data = F)
```

Model lm2: Age and Emotion as fixed and random effects in the model:

```
lm2b <- lmer(latency_cleaned ~ timepoint_recode*emotion_factor + (timepoint_recode+emotion_factor|pid), vig,
             control = lmerControl(optimizer ='optimx', optCtrl=list(method='L-BFGS-B')),
             REML = TRUE)
```

```
## Warning in optimx.check(par, optcfg$ufn, optcfg$ugr, optcfg$uhess, lower, : Parameters or bounds appear to hav
e different scalings.
##   This can cause poor performance in optimization.
##   It is important for derivative free methods like BOBYQA, UOBYQA, NEWUOA.
```

```
## boundary (singular) fit: see ?isSingular
```

```
#summary(lm2b)
#confint(lm2b, method = "Wald")

if (isSingular(lm2b)) { print(check_singularity(lm2b)) }
```

```
## pid.emotion_factorangry pid.emotion_factorhappy
##                        0                        0
```

This model above throws a warning error. This model is likely over complicated given our dataset.

Plot of predicted model lm2

```
plot_model(lm2b, type = "int", mdrt.values = "meansd", show.data = F)
```

Model lm3: Age as fixed and random, Emotion as fixed effects in model:

```
lm3b <- lmer(latency_cleaned ~ timepoint_recode*emotion_factor + (timepoint_recode|pid), vig,
             control = lmerControl(optimizer ='optimx', optCtrl=list(method='L-BFGS-B')),
             REML = TRUE)
summary(lm3b)
```

```
## Linear mixed model fit by REML. t-tests use Satterthwaite's method [
## lmerModLmerTest]
## Formula:
## latency_cleaned ~ timepoint_recode * emotion_factor + (timepoint_recode |
##     pid)
##    Data: vig
## Control: lmerControl(optimizer = "optimx", optCtrl = list(method = "L-BFGS-B"))
##
## REML criterion at convergence: 17601.9
##
## Scaled residuals:
##     Min      1Q  Median      3Q     Max
## -2.8369 -0.5795 -0.1745  0.3944  4.1936
##
## Random effects:
##  Groups   Name             Variance Std.Dev. Corr
##  pid      (Intercept)      23530    153.4
##           timepoint_recode  4330     65.8    -0.90
##  Residual                  14784    121.6
## Number of obs: 1382, groups:  pid, 260
##
## Fixed effects:
##                                     Estimate Std. Error      df t value
## (Intercept)                          540.938     15.278 420.181  35.407
## timepoint_recode                     -17.212      7.016 309.222  -2.453
## emotion_factorangry                   29.891     14.768 987.829   2.024
## emotion_factorhappy                   19.756     14.651 986.418   1.348
## timepoint_recode:emotion_factorangry  -14.082      6.625 985.205  -2.126
## timepoint_recode:emotion_factorhappy   -5.049      6.568 980.813  -0.769
##                                     Pr(>|t|)
## (Intercept)                           <2e-16 ***
## timepoint_recode                      0.0147 *
## emotion_factorangry                   0.0432 *
## emotion_factorhappy                   0.1778
## timepoint_recode:emotion_factorangry   0.0338 *
## timepoint_recode:emotion_factorhappy   0.4423
## ---
## Signif. codes:  0 '***' 0.001 '**' 0.01 '*' 0.05 '.' 0.1 ' ' 1
##
```

```
## Correlation of Fixed Effects:
##                      (Intr) tmpnt_ emtn_fctrn emtn_fctrh tmpnt_rcd:mtn_fctrn
## timepnt_rcd          -0.857
## emtn_fctrng          -0.480  0.389
## emtn_fctrhp          -0.484  0.394  0.501
## tmpnt_rcd:mtn_fctrn  0.399 -0.465 -0.837     -0.417
## tmpnt_rcd:mtn_fctrh  0.402 -0.469 -0.417     -0.835      0.498
```

```
confint(lm3b, method = "Wald")
```

```
##                                             2.5 %     97.5 %
## .sig01                                         NA         NA
## .sig02                                         NA         NA
## .sig03                                         NA         NA
## .sigma                                         NA         NA
## (Intercept)                           510.9946013 570.881519
## timepoint_recode                      -30.9621084  -3.461361
## emotion_factorangry                     0.9467108  58.835102
## emotion_factorhappy                    -8.9591467  48.471469
## timepoint_recode:emotion_factorangry -27.0669871  -1.097448
## timepoint_recode:emotion_factorhappy -17.9230425   7.824888
```

Plot of predicted model lm3

```
final<-plot_model(lm3b, type = "int", show.data = F, colors=c("grey40", "red2","limegreen"), legend.title="Emotio
n Configuration")+
  labs(x = "Assessment", y = "Latency to Fixate the Face (ms)")
final+theme_bw()
```

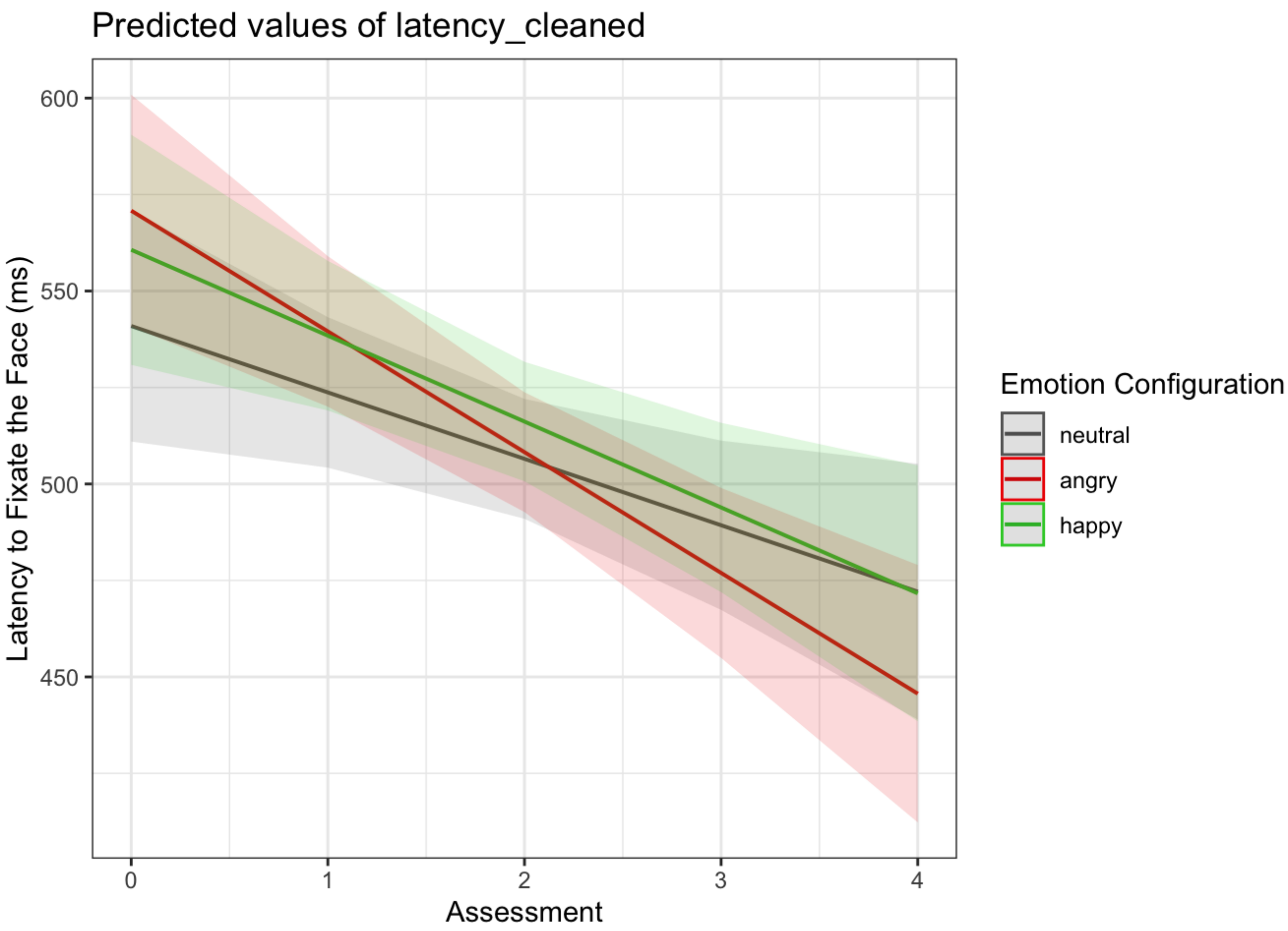

ANOVA Comparison Test of Model Fit:

```
anova(lm0b, lm1b, lm3b)
```

```
## refitting model(s) with ML (instead of REML)
```

```
## Data: vig
## Models:
## lm0b: latency_cleaned ~ 1 + (1 | pid)
## lm1b: latency_cleaned ~ timepoint_recode + (timepoint_recode | pid)
## lm3b: latency_cleaned ~ timepoint_recode * emotion_factor + (timepoint_recode | pid)
##      npar   AIC   BIC  logLik deviance    Chisq Df Pr(>Chisq)
## lm0b    3 17799 17815 -8896.5    17793
## lm1b    6 17654 17685 -8820.9    17642 151.2346  3     <2e-16 ***
## lm3b   10 17655 17708 -8817.7    17635   6.3209  4     0.1764
## ---
## Signif. codes:  0 '***' 0.001 '**' 0.01 '*' 0.05 '.' 0.1 ' ' 1
```

We selected Model 3 as the final model.

Follow-up paired samples t-test to examine when a AB to threat is present

```
t.test(reshaped_vig$neu_4M, reshaped_vig$ang_4M, paired = TRUE, alternative = "two.sided") #4-month
```

```
##
##  Paired t-test
##
## data:  reshaped_vig$neu_4M and reshaped_vig$ang_4M
## t = -0.43413, df = 56, p-value = 0.6659
## alternative hypothesis: true difference in means is not equal to 0
## 95 percent confidence interval:
##  -57.16819  36.80329
## sample estimates:
## mean of the differences
##               -10.18245
```

```
t.test(reshaped_vig$neu_8M, reshaped_vig$ang_8M, paired = TRUE, alternative = "two.sided") #8-month
```

```
##
##  Paired t-test
##
## data:  reshaped_vig$neu_8M and reshaped_vig$ang_8M
## t = -0.96094, df = 132, p-value = 0.3383
## alternative hypothesis: true difference in means is not equal to 0
## 95 percent confidence interval:
##  -37.87066  13.10651
## sample estimates:
## mean of the differences
##               -12.38208
```

```
t.test(reshaped_vig$neu_12M, reshaped_vig$ang_12M, paired = TRUE, alternative = "two.sided") #12-month
```

```
##
##  Paired t-test
##
## data:  reshaped_vig$neu_12M and reshaped_vig$ang_12M
## t = -0.61768, df = 104, p-value = 0.5381
## alternative hypothesis: true difference in means is not equal to 0
## 95 percent confidence interval:
##  -31.1586  16.3580
## sample estimates:
## mean of the differences
##               -7.400301
```

```
t.test(reshaped_vig$neu_18M, reshaped_vig$ang_18M, paired = TRUE, alternative = "two.sided") #18-month
```

```
##
##  Paired t-test
##
## data:  reshaped_vig$neu_18M and reshaped_vig$ang_18M
## t = 0.82837, df = 87, p-value = 0.4097
## alternative hypothesis: true difference in means is not equal to 0
## 95 percent confidence interval:
##  -15.20128  36.92628
## sample estimates:
## mean of the differences
##                 10.8625
```

```
t.test(reshaped_vig$neu_24M, reshaped_vig$ang_24M, paired = TRUE, alternative = "two.sided") #24-month
```

```
##
##  Paired t-test
##
## data:  reshaped_vig$neu_24M and reshaped_vig$ang_24M
## t = 2.2174, df = 51, p-value = 0.03108
## alternative hypothesis: true difference in means is not equal to 0
## 95 percent confidence interval:
##   3.448742 69.451943
## sample estimates:
## mean of the differences
##                36.45034
```

```
#means and SDs for sig t-test
mean(reshaped_vig$neu_24M, na.rm=TRUE)
```

```
## [1] 529.0749
```

```
sd(reshaped_vig$neu_24M, na.rm=TRUE)
```

```
## [1] 160.6004
```

```r
mean(reshaped_vig$ang_24M, na.rm=TRUE)
```

```
## [1] 478.1866
```

```r
sd(reshaped_vig$ang_24M, na.rm=TRUE)
```

```
## [1] 114.7062
```
